# Osmosensor-mediated control of Ca^2+^ spiking in pollen germination

**DOI:** 10.1038/s41586-024-07445-6

**Published:** 2024-05-22

**Authors:** Songyu Pei, Qi Tao, Wenke Li, Guoning Qi, Borong Wang, Yan Wang, Shiwen Dai, Qiujing Shen, Xi Wang, Xiaomei Wu, Shijian Xu, Lynn Theprungsirikul, Jingyuan Zhang, Liang Liang, Yuantao Liu, Kena Chen, Yang Shen, Bridget M. Crawford, Mengjia Cheng, Qi Zhang, Yiqi Wang, Hongli Liu, Benguang Yang, Bryan Krichilsky, Jessica Pei, Karen Song, Douglas M. Johnson, Zhonghao Jiang, Feihua Wu, Gary B. Swift, Huanghe Yang, Zhonghua Liu, Xuexiao Zou, Tuan Vo-Dinh, Feng Liu, Zhen-Ming Pei, Fang Yuan

**Affiliations:** 1 College of Horticulture and Landscape, Hunan Agricultural University, Changsha, China; 2 Department of Biology, Duke University, Durham, NC USA; 3 Fitzpatrick Institute for Photonics, Duke University, Durham, NC USA; 4 College of Life Sciences, Zhejiang University, Hangzhou, China; 5 College of Life and Environmental Sciences, Hangzhou Normal University, Hangzhou, China; 6 Fuqua School of Business, Duke University, Durham, NC USA; 7 Department of Physics, Duke University, Durham, NC USA; 8 Department of Biochemistry, Duke University, Durham, NC USA

**Keywords:** Plant signalling, Calcium signalling, Ion transport, Plant physiology

## Abstract

Higher plants survive terrestrial water deficiency and fluctuation by arresting cellular activities (dehydration) and resuscitating processes (rehydration). However, how plants monitor water availability during rehydration is unknown. Although increases in hypo-osmolarity-induced cytosolic Ca^2+^ concentration (HOSCA) have long been postulated to be the mechanism for sensing hypo-osmolarity in rehydration^1,2^, the molecular basis remains unknown. Because osmolarity triggers membrane tension and the osmosensing specificity of osmosensing channels can only be determined in vivo^3–5^, these channels have been classified as a subtype of mechanosensors. Here we identify bona fide cell surface hypo-osmosensors in *Arabidopsis* and find that pollen Ca^2+^ spiking is controlled directly by water through these hypo-osmosensors—that is, Ca^2+^ spiking is the second messenger for water status. We developed a functional expression screen in *Escherichia coli* for hypo-osmosensitive channels and identified OSCA2.1, a member of the hyperosmolarity-gated calcium-permeable channel (OSCA) family of proteins^6^. We screened single and high-order OSCA mutants, and observed that the *osca2.1/osca2.2* double-knockout mutant was impaired in pollen germination and HOSCA. OSCA2.1 and OSCA2.2 function as hypo-osmosensitive Ca^2+^-permeable channels in planta and in HEK293 cells. Decreasing osmolarity of the medium enhanced pollen Ca^2+^ oscillations, which were mediated by OSCA2.1 and OSCA2.2 and required for germination. OSCA2.1 and OSCA2.2 convert extracellular water status into Ca^2+^ spiking in pollen and may serve as essential hypo-osmosensors for tracking rehydration in plants.

## Abstract (web summary)

Screening in *Escherichia coli* and biochemical experiments show that in *Arabidopsis thaliana*, OSCA2.1 and OSCA2.2 function as plant sensors of hypo-osmolarity, utilize Ca^2+^ oscillations as second messengers and have crucial roles in pollen germination.

## Main

**Extended Data Fig. 1:**
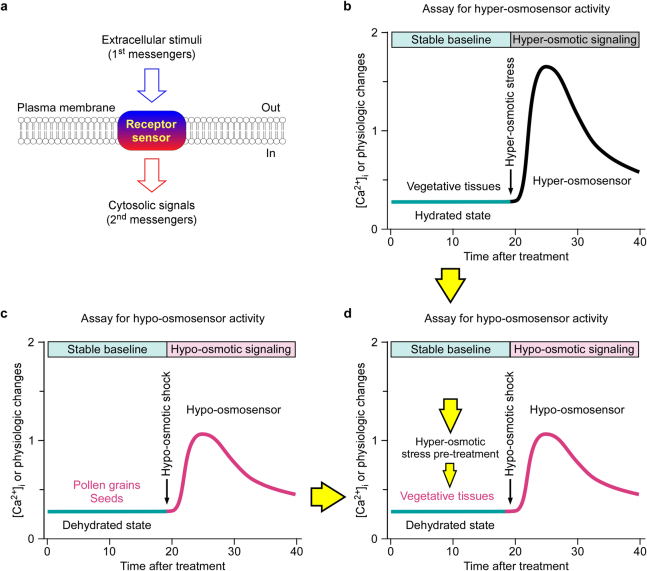
Schematic illustration of the perception of extracellular stimuli by cell-surface receptors, and the characteristics of analyzing hypo-osmosensors in plants. **a**, The extracellular stimuli (first messengers) are perceived by cell-surface receptors/sensors, and converted to cytosolic signals (second messengers). It is essential for living organisms to sense environmental signals to program accordingly growth, development and survival^1,4,5,21,23,34,46,89–94^. The environmental signals include physical, chemical, and biological stimuli, which are called the first messengers. The cytosolic signals include diffusible small molecules and ions, which are called the second messengers^89^. These receptors are usually ion channels, receptor kinases (RTKs)/receptor-like kinases (RLKs), and G-protein-coupled receptors (GPCRs) in animals and plants, and their activation often leads to increases in [Ca^2+^]_i_ through either Ca^2+^ influx across the plasma membrane or Ca^2+^ release from internal stores^4,5,23,90^. It is well known that Ca^2+^ ion serves as a versatile second messenger for various external stimuli in living organisms^1,19,21,89,90^. **b**,**c**, Comparison between the assays for the activities of hyper-osmosensors and hypo-osmosensors in plants. With respect to the analysis of cytosolic signals, for example, [Ca^2+^]_i_, the stable baseline at the resting state should be established first. Subsequently, the extracellular stimulus, such as hyper-osmotic stress (**b**) or hypo-osmotic shock (**c**), could be applied as indicated by the arrow, and the dynamic of cytosolic signals could be recorded continuously, which reflects the activity of the cell-surface receptor corresponding to the stimulus applied. These terms of “osmosensing”, “osmosensitive” and “osmosensor” have been widely used in microorganisms, plants, and animals^2,4,11,12,87^. In general, as long as cells face the amount of available water less than “normal” in the extracellular spaces, which causes cells to lose water from the cytosol, this is called as hyper-osmotic stress/stimulation. On the other hand, if cells face the amount of water more than “normal”, which leads to water influx into the cell, this is called as hypo-osmotic shock/stimulation. Note that, the definition of hyper- or hypo-osmolarity is not about the absolute values to some extent, rather the relationship to the osmolarity in the cytosol, similar to the de-polarization and hyper-polarization in relative to the resting membrane potential. Note that, under the normal growth conditions without any osmotic stress, vegetative tissues (seedlings) are well hydrated, and thus by default, the vegetative tissues are at the stable baseline of the hydrated state (**b**). Hyper-osmotic stress could be applied directly to these vegetative tissues, and cytosolic signaling processes could be recorded accordingly^6,85,87^. In contrast, also under the normal growth conditions, pollen grains and seeds are well dehydrated. By default, they are at the stable baseline of the dehydrated state, and hypo-osmotic shock could be applied directly to analyze hypo-osmosensitive activities (**c**)^41,43,48,72,95^. Apparently, without the need of any pretreatment, vegetative tissues are good systems for the analysis of hyper-osmotic stress, while pollen grains and seeds are good systems for the analysis of hypo-osmotic stress. **d**, The assay for hypo-osmotic shock-induced signaling or physiological changes in vegetative tissues is much more challenging technically. The analyses of hypo-osmotic shock-induced responses in vegetative tissues have been well described in a number of studies^16–18,84^. Essentially, these vegetative tissues have to be pre-treated with hyper-osmotic conditions for a relatively long period of time (hours or days) first to establish the stable baseline of the dehydrated state, then hypo-osmotic shock could be applied for the analysis of hypo-osmotic signaling processes (in minutes) or physiological changes (in hours and days). Therefore, it has been hard to establish Ca^2+^-imaging-based genetic screens for mutants defective in HOSCA, in contrast to mutants defective in hyper-osmotic stress-induced Ca^2+^ increases^6^. Evidently, if vegetative tissues are treated with drought stress by withdraw water supply for days to establish the dehydrated state, and then water is resupplied to allow rehydration^14^, the phenotypes of growth and survival rate could reflect the complex effects on both the dehydration and/or rehydration processes.

All living organisms, particularly sessile land plants, must monitor water in their environment to programme growth and development^3,7^. Land plants evolved from aquatic ancestors and adapted to the terrestrial environment by overcoming two seemingly insurmountable obstacles: water deficiency and fluctuation^8^. To survive dry environments and harsh seasons, ancestral land plants not only acquired specific structures for water acquisition, transport and management, but also improved developmental strategies. One of their most effective strategies is to arrest cellular activities via water loss to form drying or desiccation-tolerant structures, such as spores, pollen and seeds^8–10^. Rehydration is likely to occur via hypo-osmosensing processes that resemble cell surface hypo-osmosensors in other organisms^4,11,12^ and involve second messenger systems (Extended Data Fig. 1a)—that is, the lower osmolarity in extracellular spaces relative to cytosol is tracked and converted to a second messenger to initiate biological activities. However, the plant hypo-osmosensor remains unidentified^13,14^.

More than 35 years ago, Tazawa et al. observed a Ca^2+^ requirement for cell turgor regulation in algae^2^, and recorded HOSCA using injected Ca^2+^-sensitive bioluminescent aequorin^15^, which was later confirmed in algae using injected fluorescence dyes^16,17^ and in aequorin-expressing tobacco^18^. HOSCA has long been hypothesized to be a hypo-osmolarity perceiving mechanism, as Ca^2+^ acts as a second messenger for various stimuli^19–21^ (Extended Data Fig. 1a–c). Indeed in animals, some transient receptor potential (TRP) channels—a family of around 30 sensory Ca^2+^-permeable channels for diverse stimuli—function as hypo-osmosensors^5,12,22,23^. Nevertheless HOSCA-associated molecular components remain unidentified in plants, partially owing to technical challenges of isolating them (Extended Data Fig. 1b–d).

To demonstrate a function for HOSCA in sensing of hypo-osmolarity (hypo-osmosensing), several criteria must be met. First, the HOSCA mutant must exhibit defective HOSCA. Second, the physiological processes in response to hypo-osmotic treatment must be compromised in HOSCA mutants. Third, if the HOSCA encodes an ion channel, the channel must be gated by hypo-osmolarity in planta. We previously used aequorin Ca^2+^ imaging-based genetic screens to identify the cell surface sensors for hyper-osmotic stress (OSCA1 (ref. ^6^)), salt stress (GIPC^24^) and H_2_O_2_ (HPCA1 (ref. ^25^)). Similarly, the receptors for ATP, lipopolysaccharide and quinone have also been indentified^26–28^. In contrast to these stimuli, for HOSCA measurements, vegetative tissues must be treated first with hyper-osmotic stress, and then with hypo-osmotic shock, which causes enormous variations of Ca^2+^ increases (Extended Data Fig. 1d), precluding Ca^2+^-dependent genetic screens. Conversely, given that several OSCAs have been identified as hyper-osmolarity-gated Ca^2+^-permeable channels^6,29–32^—that is, hyper-osmosensors—it is possible that some of the 15 OSCA family members in *Arabidopsis* could function as hypo-osmosensors. In eukaryotes, mechanosensing channels include TRPs, epithelial sodium channels, two-pore domain potassium channels, MscS-like channels (MSLs), piezo channels and OSCAs^4–6,23,33–35^. There are no plant homologues of TRP, epithelial sodium channels or two-pore domain potassium channels, whereas they do express MSLs, piezo channels and OSCAs^13,20,35^. Most MSLs are anion channels that are located in endomembranes and regulate organelle size^33^, and piezo is also localized to endomembranes^36,37^. In addition, MCA1 and MCA2 function as Ca^2+^-permeable mechanosensitive channels in roots^38^. Structural analyses of OSCAs have shown the importance of lipid interactions and conformational changes in their activation, and some OSCAs can be activated by hyper-osmotic treatments and mechanical poke or negative pressure to the plasma membrane when analysed in heterologous expression systems^6,29–32^, but they have not been demonstrated to function as hypo-osmosensors in planta. However, the expression systems that have been used to analyse other hypo-osmosensors, such as animal cells for TRPV4 and *E*. *coli* for MscS and MscL mechanosensitive channels^22,39^, have not been used to screen OSCAs stringently for their hypo-osmosensing activities.

During our initial cloning of OSCAs, we found that they were toxic to *E*. *coli* and that the toxicity was suppressed by increasing the osmolarity of the medium, suggesting that the toxicity might be caused by hypo-osmosensitive activities. Here we developed a robust growth screen in *E*. *coli* to systematically analyse this subtle phenotype, identified OSCAs as hypo-osmosensors, revealed their activities responsible for HOSCA in *Arabidopsis* pollen, and identified pollen Ca^2+^ oscillations as the second messenger that enables apoplastic osmolarity to initiate germination.

## Screening for hypo-osmosensitive OSCAs

The lack of molecular information on HOSCA prompted us to use established approaches to identify hypo-osmosensing channels, but all attempts unfortunately failed. As TRPV4 is a hypo-osmosensor^22^, we used expression cloning in human embryonic kidney 293 (HEK293) cells. We sought to develop Ca^2+^-genetic screens for HOSCA mutants. We expressed 15 OSCAs in HEK293 cells but observed mixed channel specificities that did not distinguish between hypo-osmotic shock and mechanical stress. We expressed 15 OSCAs in the *E. coli* 7-MscL/MscS-knockout strain^39^. We also tested individual transfer DNA (T-DNA) mutants of 15 OSCAs, but could not identify phenotypes with reduced HOSCA. Eventually, we followed the assumption that OSCAs could encode both hyper- and hypo-osmosensing channels. During our initial cloning of OSCAs into *E*. *coli* vectors, we encountered difficulties, possibly owing to their toxicity^40^. Because excess Na^+^ ions might permeate through OSCA channels, we grew the *E. coli* cells with reduced NaCl in the medium to clone several OSCAs into the vectors, but some of these OSCAs could not be cloned until the osmolarity was increased. We speculated that these OSCAs may be active and toxic under low osmolarity, which motivated us to conduct thorough analyses on these channels.

**Fig. 1:**
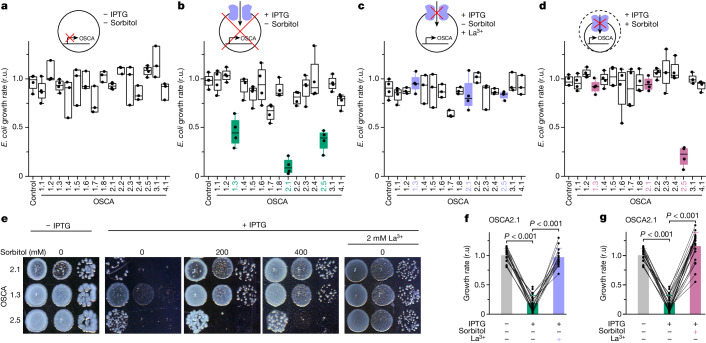
Identification of hypo-osmosensitive OSCA2.1 by a functional expression screen in *E*. *coli.* **a**–**d**, Growth rates of *E*. *coli* carrying vectors for expression of 15 *Arabidopsis* OSCA family proteins on low-salt hypo-osmotic medium (70 mOsm) without (**a**) or with IPTG induction (**b**), or with IPTG and LaCl_3_ (**c**) or 200 mM sorbitol (270 mOsm; **d**). Red crosses indicate lack of OSCA expression (**a**), toxicity to cells (**b**) and inhibition of OSCA channels (**c**,**d**). **d**, The dashed circle indicates the relative cell size before hyper-osmotic treatment. OSCA1.3, OSCA2.1 and OSCA2.5 are highlighted in **b**–**d**. Growth rates of controls without IPTG were arbitrarily set to 1. In box plots, the centre line is the median, box edges delineate first and third quartiles and whiskers extend to minimum and maximum values (*n* = 3 or 4 independent experiments). r.u., relative unit. **e**–**g**, Side-by-side assay of *E. coli* expressing OSCAs as in **a**–**d** (**e**) and growth rates of *E. coli* expressing OSCA2.1 in 2 mM LaCl_3_ (**f**) or 200 mM sorbitol (**g**). Data are mean ± s.d. (*n* = 3 or 4 independent experiments). Source Data

**Extended Data Fig. 2:**
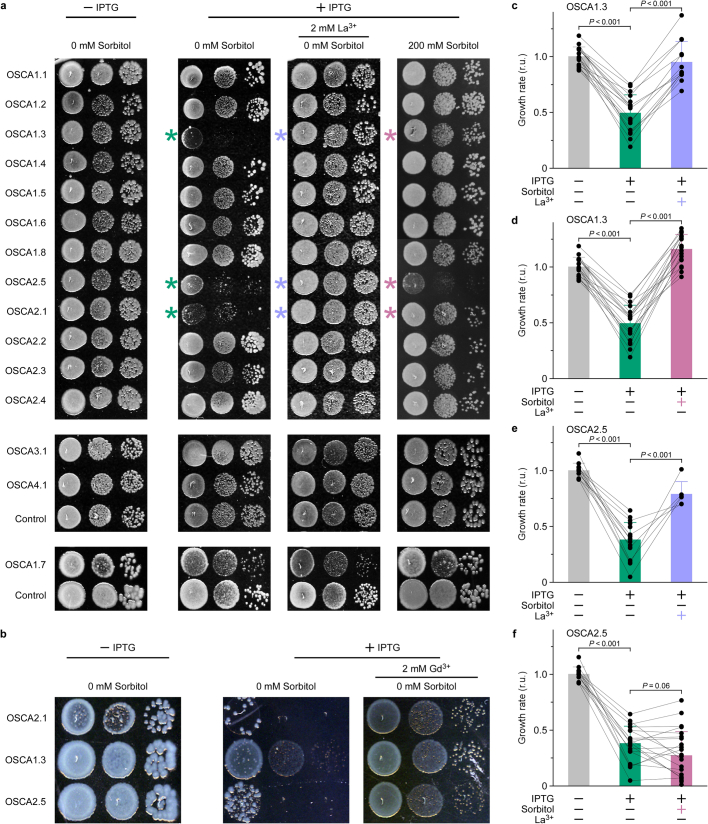
hypo-osmosensitive OSCA2.1 and OSCA1.3 are identified through functional expression screen in *E*. *coli.* **a**, Images show *E. coli* cells expressing 15 OSCA genes after 16 h of growth on the low salt hypo-osmotic Lysogeny broth (LSHypo-LB) agar media (5 mM NaCl, 3 mM KCl; 70 mOsm) in the absence or presence of IPTG induction. Under the IPTG induction, 2 mM LaCl_3_ (a Ca^2+^ channel blocker) or 200 mM sorbitol (for elevated osmolarity, 270 mOsm) were added to the media. The growth rates of similar experiments were analyzed and shown in Fig. 1a–d. *, *E. coli* cells expressing OSCAs with the reduced growth under LSHypo-LB agar media. **b**, Side-by-side assay of *E. coli* cells expressing OSCA2.1, OSCA1.3 and OSCA2.5 in the presence or absence of Ca^2+^ channel blocker Gd^3+^ (2 mM GdCl_3_) in experiments similar to these in **a**. Gd^3+^ restored the *E. coli* growth, suggesting that similar to La^3+^, Gd^3+^ could block OSCA channel activities to alleviate their toxicity. **c**,**d**, Growth rates of *E. coli* cells expressing hypo-osmosensitive OSCA1.3 in the presence of LaCl_3_ (**c**) or 200 mM sorbitol (**d**) from experiments similar to these in Fig. 1e. Note that, OSCA1.3 was less toxic to *E. coli* than OSCA2.1 when expressed under hypo-osmotic conditions, suggesting that OSCA2.1 might be more hypo-osmosensitive than OSCA1.3 under the imposed conditions. Quantifications of growth rates are shown (mean ± s.d.; *n* = 3, 4 or 5 independent experiments). **e**,**f**, Quantification of growth rates of *E. coli* cells expressing the toxic OSCA2.5 from experiments similar to these in Fig. 1e. The *E. coli* growth was rescued by LaCl_3_, suggesting that the toxicity of OSCA2.5 was caused by its Ca^2+^-permeable channel activity (**e**); but was not rescued by the hyper-osmotic treatment (200 mM sorbitol; **f**), indicating that OSCA2.5 was activated by factors other than the hypo-osmolarity. Data are mean ± s.d. (*n* = 3 or 4 independent experiments). Note that, using this stringent growth screen, we could only identify these hypo-osmosensitive OSCAs, whose hypo-osmosensitive channel activities were expressed to a level toxic to *E. coli*, but could not rule out others having hypo-osmosensitivities, because their activities did not rise to the toxic threshold, possibly due to low protein expression levels. Source Data

In the low-salt hypo-osmotic medium, *E*. *coli* clones encoding 15 different OSCAs grew normally without isopropyl β-d-1-thiogalactopyranoside (IPTG) induction (Fig. 1a and Extended Data Fig. 2a). In the presence of IPTG, cells expressing OSCA2.1, OSCA2.5 and OSCA1.3 grew more slowly (Fig. 1b), and the Ca^2+^ channel inhibitor La^3+^ restored growth (Fig. 1c). Increases in osmolarity of the medium rescued growth for cells expressing OSCA2.1 and OSCA1.3, but not those expressing OSCA2.5 (Fig. 1d). Direct comparison of these cells confirmed that OSCA2.1, OSCA1.3 and OSCA2.5 were toxic under hypo-osmotic conditions and that the Ca^2+^ channel inhibitors La^3+^ and Gd^3+^ blocked the toxicity of all three channels, whereas sorbitol suppressed the toxicity of OSCA2.1 and OSCA1.3 only (Fig. 1e–g and Extended Data Fig. 2b–f). These results suggest that OSCA2.1 and OSCA1.3 may be hypo-osmosensitive channels, whereas OSCA2.5 in likely to have a distinct physiological activation mechanism.

## OSCAs are essential for pollen germination

Receptors convert an external signal into a second messenger (Extended Data Fig. 1a) to regulate downstream physiological processes. We therefore tested whether hypo-osmosensitive OSCAs are required for processes that are known to be regulated by water availability. Pollen germination and seed germination are essential processes for fitness and survival under extreme water regimes^7,8,10,14^, in which cells emerge from a desiccated state to initiate biological activities in response to increases in water availability (Extended Data Fig. 1c). Single-cell haploid pollen provide a useful model for studying plant responses to external signals^9,10,41^, whereas seed germination is involved in the coordination of multiple cell types.

**Fig. 2:**
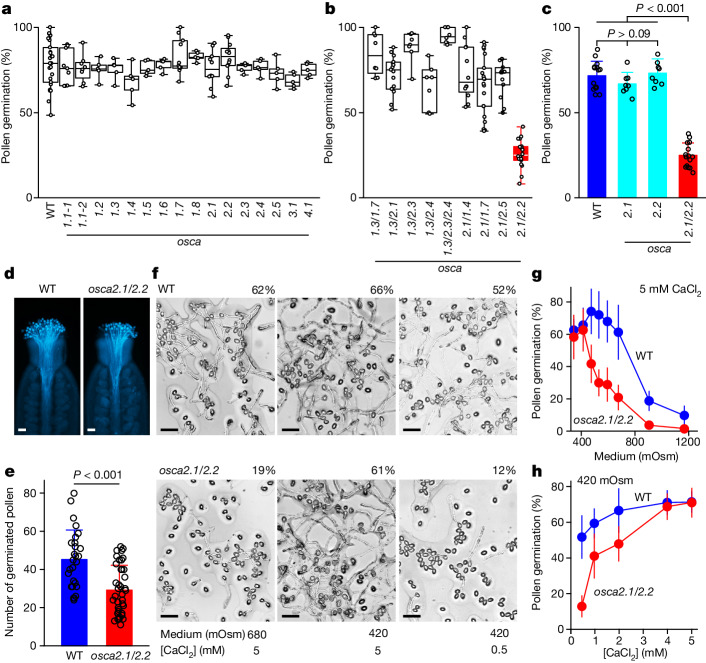
OSCA2.1 and OSCA2.2 are required for water-initiated and Ca^2+^-dependent pollen germination in *Arabidopsis.* **a**,**b**, Pollen germination rates in *Arabidopsis* single OSCA T-DNA mutants (**a**) and double or triple mutants (**b**). *n* = 3–5 independent experiments. In box plots, the centre line is the median, box edges delineate first and third quartiles and whiskers extend to minimum and maximum values. WT, wild type. **c**, Pollen germination rates in OSCA2.1 and OSCA2.2 single and double mutants placed side-by-side on the standard germination medium (535 mOsm) for 6 h, as in Extended Data Fig. 3c. Data are mean ± s.d.; *n* = 3–5 independent experiments. **d**,**e**, Micrographs of pollen grains from wild-type and *osca2.1/2.2* plants placed on wild-type stigmas and stained with aniline blue. Pollen grains were stained 2 h after pollination (**d**) and germination rates were counted (**e**). Data are mean ± s.d.; WT, *n* = 27 stigmas; *osca2.1/2.2*, *n* = 39 stigmas. Scale bars, 50 μm. **f**–**h**, Defects of hypo-osmolarity-dependent and Ca^2+^-dependent germination in *osca2.1/2.2* pollen. Pollen grains were placed on agarose medium with high or low osmolarity (relative to 535 mOsm) and high or low Ca^2+^ concentration (relative to 2 mM) for 6 h, and were viewed under the microscope (**f**). **f**, Germination rates are indicated as a percentage above each image. Scale bars, 50 μm. **g**,**h**, Germination rates with 5 mM CaCl_2_ and varying osmolarity (**g**) or low osmolarity (420 mOsm) and varying CaCl_2_ concentration (**h**) in agarose media from experiments similar to **f**. Data are mean ± s.d.; *n* = 3 independent experiments for each data point; two-way analysis of variance (ANOVA), *P* < 0.001. Source Data

**Extended Data Fig. 3:**
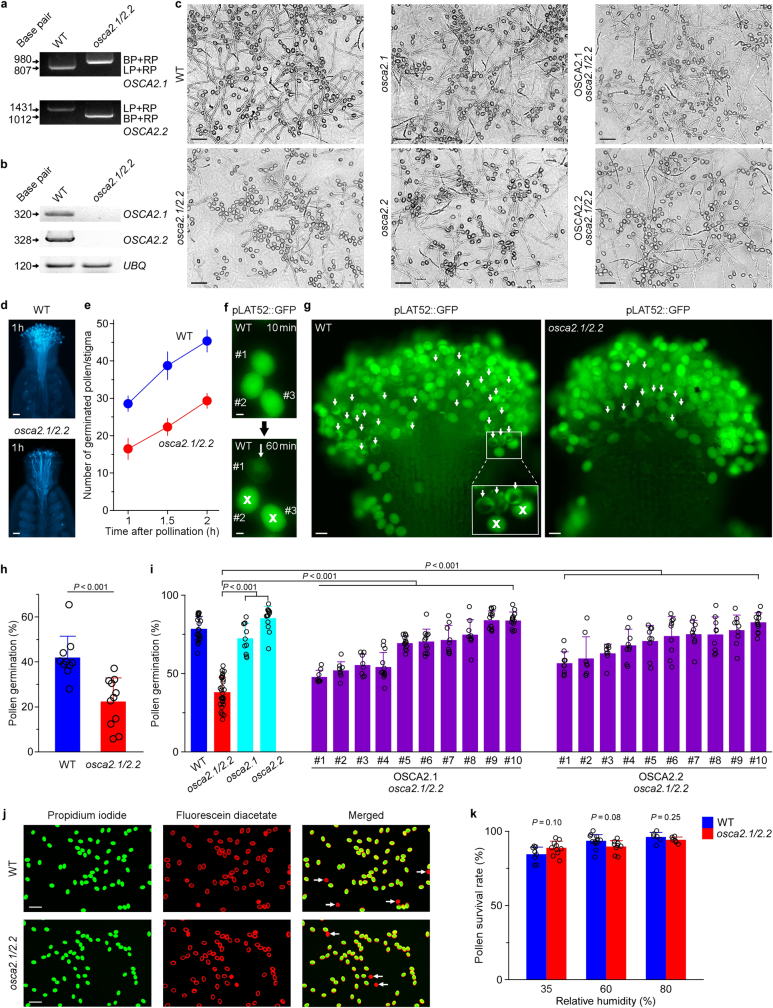
The hypo-osmosensitive OSCA2.1 and its close homologue OSCA2.2 are both required for pollen germination. **a**,**b**, Genotyping and gene expression analysis of the *osca2.1/2.2* double-knockout mutant. PCR reactions showed a flanking DNA fragment upstream (LP) and downstream of the insertion site (RP) in wild type (WT) but not in the *osca2.1/2.2* double mutant, and a DNA fragment flanking the T-DNA border (BP) and the downstream of the insertion site (RP) in *osca2.1/2.2* but not WT, which demonstrated that *osca2.1/2.2* is a homozygous double T-DNA insertion line (**a**). The mRNAs of OSCA2.1 and OSCA2.2 were absent in the *osca2.1/2.2* double mutant, indicating that *osca2.1/2.2* is a knock-out/null mutant (**b**). Similar results were seen in > 3 times. **c**, Representative micrographs of pollen grains from wild type, the *osca2.1* and *osca2.2* single mutants, the *osca2.1/2.2* double mutant, and the *OSCA2.1 osca2.1/2.2* and *OSCA2.2 osca2.1/2.2* complementation lines, which were placed on the standard germination media (535 mOsm) for 6 h. Similar experiments were carried out and shown in Fig. 2c. The complementation lines are these #9 or #10 lines that were described below in **i**. Note that, although OSCA2.1 and 2.2 were both hypo-osmosensitive channels and redundant in pollen germination, the reason that only OSCA2.1 was identified in the *E. coli* assay (Fig. 1) was that the ion-transporting activity of OSCA2.2 hypo-osmosensitive channels was not expressed functionally to the toxic threshold as compared to that of OSCA2.1, possibly due to low protein expression of OSCA2.2. Conversely, although OSCA1.3 hypo-osmosensitivity was identified in *E. coli*, the reason that the *osca1.3/2.1* double mutant did not show a pollen germination defect as compared to *osca2.1/2.2* (Fig. 2b) might be that the activity of OSCA1.3 was probably lower than that of OSCA2.2 in pollen, and knocking out OSCA1.3 together with OSCA2.1 could not lower the activity to a threshold sufficient to disrupt germination. Scale bars are 50 μm. Similar results were seen in > 3 times. **d**–**e**, Analysis of time course of pollen germination on wild-type stigmas via aniline blue staining. The flowers from wild-type plants were emasculated 24 h prior to pollination, and pollen grains from wild type and *osca2.1/2.2* plants were dabbed onto pre-emasculated stigmas. After 1, 1.5, and 2 h of pollination, stigmas with pollen grains were stained with the aniline blue, fluorescence images were taken, and the numbers of pollen grains that stayed on the stigma were counted. Representative micrographs of pollen grains from wild type and *osca2.1/2.2* taken at 1 h after pollination (**d**) as well as at 2 h (Fig. 2d) are shown. Scale bars are 50 μm. The numbers of pollen grains from these experiments were plotted as the function of time after pollination (**e**; mean ± s.e.; *n*_WT_ = 23, 13, 27 stigmas for each data point, *n*_*osca2.1/2.2*_ = 9, 13, 39 stigmas for each data point; two-way ANOVA). With increases in the germination time, the numbers of germinated pollen grains increased in both wild type and *osca2.1/2.2*, but consistently less germinated pollen grains in *osca2.1/2.2*, suggesting that *osca2.1/2.2* was defective in pollen germination under the imposed in vivo condition, i.e. at a given osmolarity at the stigma. Similar results were seen in > 5 times. **f**, Analysis of changes in GFP fluorescence during in vitro pollen germination. Pollen grains from wild-type plants constitutively expressing GFP, which was driven by a pollen specific promoter LAT52, were placed on the standard in vitro germination media, and GFP fluorescence images were taken every 30 s for 300 min. Representative images taken for the same three wild-type pollen grains before germination (top panel) at the time of 10 min and after one pollen grain germinated (bottom panel) at the time about 100 min are shown here. Similar results were seen in > 5 times. The three wild-type pollen grains were labeled as #1, #2 and #3. The #1 wild-type grain germinated and was indicated by an arrow. The #2 and #3 WT grains did not germinate and were labeled by “x”. After germination, the #1 grain displayed low fluorescence with a circular pattern, while the fluorescence of other two non-germinated #2 and #3 grains displayed strong fluorescence. This phenotype of changes in GFP fluorescence during germination was used to score germinated vs non-germinated pollen grains for the assay of in vivo pollen germination on stigma in **g**,**h**. Scale bars are 5 μm. **g**–**h**, Analysis of pollen germination on WT stigmas using pollen grains constitutively expressing GFP. As described in experiments in Fig. 2d,e, for in vivo pollen germination on stigma, the flowers from wild-type plants (without GFP expression) were emasculated 24 h prior to pollination, and pollen grains from wild-type and *osca2.1/2.2* plants (with GFP expression as in **f**) were dabbed onto the surfaces of pre-emasculated stigmas. After 30 min of pollination, stigmas with pollen grains were analyzed, and GFP fluorescence images were collected (**g**). Scale bars are 20 μm. Based on images in **f**, germinated pollen grains displayed weaker GFP fluorescence, notably much weaker in the center of pollen grains, and were labeled with arrows. In contrast, the non-germinated grains displayed solid fluorescence patterns, which were labeled with x (see the insert). Germination rates were calculated, and data from more than 5 independent experiments were shown (**h**; mean ± s.d., *n*_WT_ = 10 stigmas, *n*_*osca2.1/2.2*_ = 11 stigmas). **i**, Complementation of the pollen germination defects in *osca2.1/2.2* by genomic DNA of OSCA2.1 or OSCA2.2 under the standard germination media (535 mOsm). The *osca2.1/2.2* mutant was the double T-DNA insertion homozygous line, and genomic segments containing their promoter and the gene were introduced into *osca2.1/2.2* (*OSCA2.1 osca2.1/2.2* or *OSCA2.2 osca2.1/2.2*). More than 100 transforms were selected, and ten representative individual transgenic lines (out of > 50), which were sorted by their germination rates (#1 to #10), were showed to have higher germination rates than *osca2.1/2.2* (mean ± s.d.; *n* = 3 independent experiments). Note that, based on their wild-type-like germination rates, the #9 and #10 lines were selected for further analysis, such as for pollen germination in **c**. These #9 and #10 lines were also used for the generation of GCaMP6-expression lines (*OSCA2.1 osca2.1/2.2* GCaMP6 or *OSCA2.2 osca2.1/2.2* GCaMP6). **j**,**k**, No difference in variability between wild-type and *osca2.1/2.2* pollen grains. Micrographs showed the viability of wild-type and *osca2.1/2.2* pollen grains after hydration (**j**). Scale bars are 50 μm. Pollen grains were stained with fluorescein diacetate and propidium iodide dyes for viable and unviable pollen grains, respectively, after incubation for 30 min on the 420 mOsm germination media. Note that, these pollen grains with the cytosol stained by fluorescein diacetate were pointed by arrows and considered unviable. Viability rates of WT and *osca2.1/2.2* pollen grains from plants grown under low (35%), normal (60%), or high relative humidity (80%) in the growth chamber were quantified (**k**). Under these growth conditions, the viability of *osca2.1/2.2* pollen grains was not affected also. Data are presented as mean ± s.d. (*n* = 3 or 4 independent experiments). Source Data

We first screened *Arabidopsis* lines mutagenized by T-DNA insertion of 15 OSCAs using an in vitro pollen germination assay, but found no significant phenotypes (Fig. 2a). On the basis of the expression of OSCA2.1, OSCA1.3 and OSCA2.5 in *E. coli*, we generated double and triple mutants, and observed lower germination rates in the *osca2.1/osca2.2* (hereafter *osca2.1/2.2*) double-knockout mutant (Fig. 2b and Extended Data Fig. 3a,b). This phenotype was verified by direct comparison of *osca2.1* and *osca2.2* single and double mutants (Fig. 2c and Extended Data Fig. 3c). The *osca2.1/2.2* pollen exhibited less germination on stigmas than wild type, as shown by aniline blue staining and GFP fluorescence imaging (Fig. 2d,e and Extended Data Fig. 3d–h), showing that OSCA2.1 and OSCA2.2 (OSCA2.1/2.2) have physiologically important roles. OSCA2.1 and OSCA2.2 expression through their endogenous promoters complemented *osca2.1/2.2* germination defects almost completely (Extended Data Fig. 3c,i), demonstrating that OSCA2.1 and OSCA2.2 functioned redundantly during pollen rehydration without altering pollen viability (Extended Data Fig. 3j,k).

## Regulation of pollen germination via OSCAs

**Extended Data Fig. 4:**
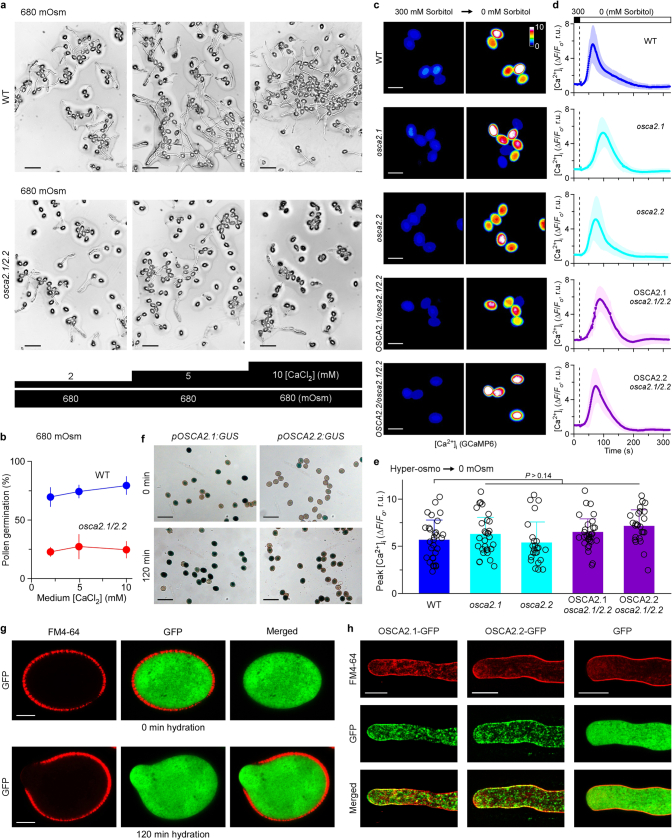
Medium Ca^2+^ effects on pollen germination, and the subcellular localization of OSCA2.1/2.2 and their requirement for hypo-osmolarity-induced [Ca^2+^]_i_ increases (HOSCA) in pollen grains. **a**,**b**, Representative micrographs of pollen grains from wild-type and *osca2.1/2.2* plants (**a**). Pollen grains were placed on the hyper-osmotic solid germination media (680 mOsm) containing variable Ca^2+^ concentrations of 2 mM, 5 mM, or 10 mM CaCl_2_ (black bars at the bottom) for 6 h, and micrographs were taken. Scale bars are 50 μm. Germination rates of wild-type and *osca2.1/2.2* pollen grains were quantified (**b**). Data are presented as mean ± s.d. (*n* = 3 trails). Similar results were seen in > 10 times. **c**–**e**, hypo-osmolarity-induced [Ca^2+^]_i_ increases (HOSCA) in pollen grains were not affected in the *osca2.1* and *osca2.1* single mutants and the *osca2.1/2.2* complementation lines (*OSCA2.1 osca2.1/2.2* and *OSCA2.2 osca2.1/2.2*). Pollen grains expressing the Ca^2+^ indicator GCaMP6 were hydrated in the germination media containing 300 mM sorbitol for 1 h, and GCaMP6 fluorescence images were taken every 1 s after the treatment with the germination solution containing 0 mM sorbitol, similar to these experiments in Fig. 3a,b. Representative GCaMP6 images scaled by a pseudo-color bar before and after hypo-osmotic treatment were shown (**c**). Scale bars are 20 μm. HOSCA in wild-type, *osca2.1*, *osca2.2*, *OSCA2.1 osca2.1/2.2* and *OSCA2.2 osca2.1/2.2* pollen grains from experiments as in **c** were analyzed for kinetics, respectively (**d**), and for peak changes in HOSCA (**e**). Data were mean ± s.d. (*n* = 3 independent experiments). **f**, *OSCA2.1-GUS* and *OSCA2.2-GUS* expression levels in pollen grains from plants expression *pOSCA2.1::OSCA2.1-GUS* and *pOSCA2.2::OSCA2.2-GUS*, respectively, were placed on the common germination media at 0 min and 120 min, and the GUS assay was performed. Scale bars are 50 μm. **g**, Pollen grains over-expressing GFP alone (*pLAT52::GFP*) were placed on the standard germination media at 0 min and 120 min, respectively, and GFP fluorescence was analyzed using confocal microcopy. *pLAT52::GFP* was used as a control for *pOSCA2.1::OSCA2.1-YFP* and *pOSCA2.2::OSCA2.2-YFP* localization in pollen grains in Fig. 3f. Scale bars are 5 μm. **h**, The plasma-membrane localization of OSCA2.1/2.2 in pollen tubes. The fluorescence of *pOSCA2.1::OSCA2.1-YFP*, *pOSCA2.2::OSCA2.2-YFP*, or FM4-64 fluorescence was analyze using confocal microscopy in newly developed pollen tubes from experiments similar to these in Fig. 3f. Merged images showed FM4-64 red fluorescence was well associated with OSCA2.1-GFP or OSCA2.2-GFP fluorescence in both the cell surface and the cytosol, but not GFP-alone fluorescence, suggesting that OSCA2.1-GFP and OSCA2.2-GFP were localized to the plasma membrane, and underwent typical endocytosis, which was seen in pollen tube tips^43,71–73^. Scale bars are 10 μm. Source Data

It has long been known that Ca^2+^ is a requirement for pollen germination^42^. We reasoned that given that OSCA2.1 and OSCA2.2 might convey extracellular hypo-osmotic information to Ca^2+^ signalling, if *osca2.1/2.2* cells were unable to track water status, decreases in medium osmolarity could overcome their germination defects. Given that sucrose serves as both a carbon source and an osmolyte, we prepared germination medium with a constant 300 mM sucrose—resulting in osmolarity lower than that of standard medium^9,10,41^ (535 mOsm)—and varied the osmolarity using sorbitol. At high osmolarity, *osca2.1/2.2* pollen germinated at lower rates than the wild type (Fig. 2f, left), and at low osmolarity, *osca2.1/2.2* germinated at similar rates to the wild type (Fig. 2f, middle). However, *osca2.1/2.2* pollen did not germinate when the Ca^2+^ concentration was decreased from the standard 5 mM to 0.5 mM (Fig. 2f, right). The synergistic effect of osmolarity and Ca^2+^ showed that *osca2.1/2.2* mutant pollen is less sensitive to decreases in osmolarity than wild type (Fig. 2g), and lowering Ca^2+^ concentration exacerbated this phenotype (Fig. 2h). Increased Ca^2+^ alone did not rescue the mutant phenotype (Extended Data Fig. 4a,b). These data show that OSCA2.1 and OSCA2.2 have an essential role in the regulation of pollen germination by medium Ca^2+^ calcium concentration^41–43^.

## OSCAs are vital for hypo-osmotic Ca^2+^ spiking

**Fig. 3:**
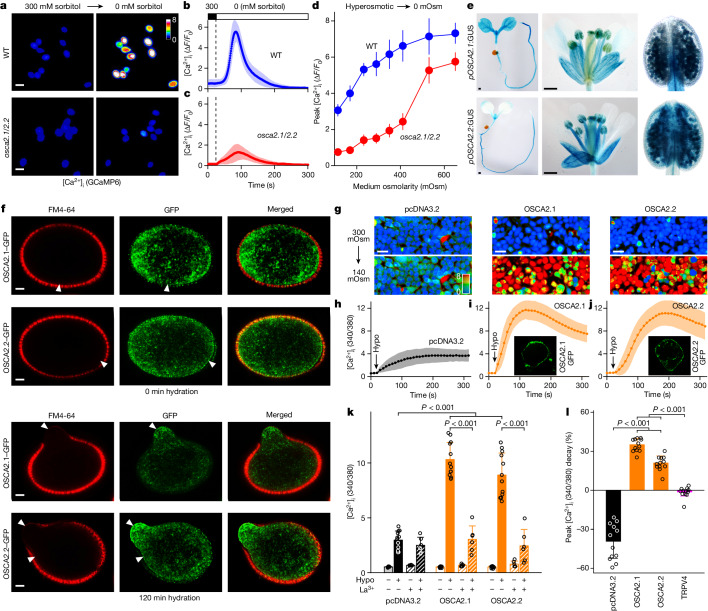
OSCA2.1 and OSCA2.2 function as hypo-osmosensitive Ca^2+^-permeable channels in pollen grains and HEK293 cells. **a**–**c**, Defects of HOSCA in *osca2.1/2.2* pollen grains. Pollen expressing GCaMP6 were hydrated in the germination medium with 300 mM sorbitol for 1 h, and fluorescence images were taken every 1 s after being treated with germination solution. **a**, The pseudocolour bar indicates relative fluorescence intensity. Scale bars, 20 μm. HOSCA in wild-type (**b**) and *osca2.1/2.2* (**c**) pollen from experiments as in **a**. Data are mean ± s.d.; WT, *n* = 71 grains; *osca2.1/2.2*, *n* = 41 grains; two-way ANOVA, *P* < 0.001. **d**, HOSCA plotted as a function of the osmolarity of pre-incubation media from experiments similar to **a**. Data are mean ± s.d.; *n* = 5 independent experiments; two-way ANOVA, *P* < 0.001. **e**, Expression patterns of *OSCA2.1*:β-glucuronidase (GUS) and *OSCA2.2*:GUS. Scale bars, 0.5 mm. **f**, Plasma membrane localization of OSCA2.1 and OSCA2.2 in pollen. OSCA2.1–GFP, OSCA2.2–GFP and FM4-64 fluorescence in pollen grains placed on germination medium for 0 and 120 min, respectively, were analysed by confocal microscopy. Arrowheads indicate the plasma membrane. Scale bars, 2 μm. **g**–**k**, OSCA2.1 and OSCA2.2 form hypo-osmosensitive Ca^2+^-permeable channels in HEK293 cells. [Ca^2+^]_i_ increases in cells expressing empty vector (pseudocolour), OSCA2.1 or OSCA2.2 were analysed by Fura-2 emission ratios (indicated by pseudocolour bar) (**g**). Cells were incubated in standard bath solution and then treated with hypo-osmotic solution (hypo). Scale bars, 20 μm. **h**–**j**, Quantification of [Ca^2+^]_i_ for empty vector (**h**), OSCA2.1 (**i**) and OSCA2.2 (**j**). Data are mean ± s.d.; *n* = 60 cells. Insets show a HEK293 cell expressing OSCA2.1–GFP (**i**) or OSCA2.2–GFP (**j**). **k**, Peak [Ca^2+^]_i_ increases from data in **h**–**j** and Extended Data Fig. 5f–i. Data are mean ± s.d.; *n* = 3 independent experiments. Hypo−, 300 mOsm; Hypo+, 140 mOsm. **l**, The decay of peak [Ca^2+^]_i_ calculated from data in **h**–**j** and Extended Data Fig. 5c,d. Data are mean ± s.d.; *n* = 12 regions of interest. Source Data

To examine HOSCA in planta^16–18^ (Extended Data Fig. 1c,d), we generated transgenic lines expressing the GFP-based Ca^2+^ indicator GCaMP6 (ref. ^44^). We placed pollen grains from these lines on agarose medium containing 300 mM sorbitol for 1 h, and then treated them with hypo-osmotic solutions. Hypo-osmotic shock did not induce increases in intracellular Ca^2+^ concentration ([Ca^2+^]_i_) in *osca2.1/2.2* pollen (Fig. 3a–c and Supplementary Video 1). HOSCA was not affected in *osca2.1* or *osca2.2* single mutants, and the HOSCA phenotypes of *osca2.1/2.2* mutant pollen could be rescued by OSCA2.1 or OSCA2.2 (Extended Data Fig. 4c–e). Increasing the osmolarity of the medium used to pretreat the pollen intensified HOSCA in both *osca2.1/2.2* and wild type, but the effect was much weaker in *osca2.1/2.2* pollen (Fig. 3d).

To further pinpoint molecular mechanisms and physiological functions of OSCA2.1 and OSCA2.2, we determined their expression patterns and subcellular localization. In GUS-reporter transgenic plants, OSCA2.1 and OSCA2.2 were expressed in whole seedlings, flowers and pollen (Fig. 3e) and expression was increased during pollen hydration (Extended Data Fig. 4f). OSCA2.1–GFP and OSCA2.2–GFP accumulated at the cell surface before hydration and at germination, and also distributed in the cytosol, possibly in preparation to form new plasma membrane (Fig. 3f), similar to MSL8 (ref. ^45^). OSCA2.1–GFP and OSCA2.2–GFP were also targeted to the cell surface and appeared to undergo exocytosis and endocytosis in newly developed pollen tubes, whereas GFP remained in the cytosol (Extended Data Fig. 4g,h).

## OSCAs form hypo-osmosensing Ca^2+^ channels

**Extended Data Fig. 5:**
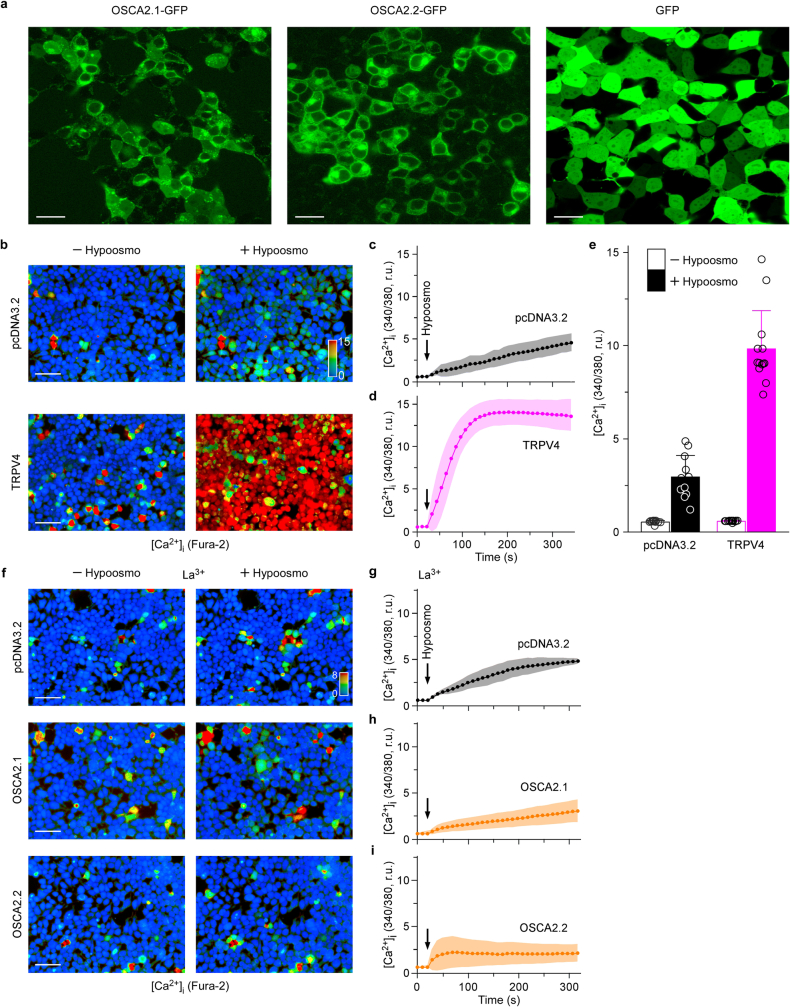
OSCA2.1 and OSCA2.2 form hypo-osmolarity-gated Ca^2+^-permeable channels in HEK293 cells. **a**, The cell-surface localization of OSCA2.1 and OSCA2.2 in HEK293 cells. HEK293 cells were transiently transfected with OSCA2.1–GFP or OSCA2.2–GFP plasmids with the GFP plasmids as a control, and GFP fluorescence was analyzed by confocal microscopy. The GFP fluorescence of OSCA2.1 and OSCA2.2 was seen in the vicinity of the plasma membrane, while the GFP alone was seen throughout the cell. Similar data were shown in the insets in Fig. 3i,j. Scale bars are 20 μm. **b**–**e**, [Ca^2+^]_i_ increases in response to hypo-osmolarity treatment in HEK293 cells expressing empty vector pcDNA3.2 or the positive control of TRPV4. The cells were incubated in standard bath solution (300 mOsm; − hypo-osmo) and then treated with the hypo-osmotic solution (140 mOsm; + hypo-osmo), and [Ca^2+^]_i_ increases were analyzed by Fura-2 emission ratios (F_340_/F_380_) and scaled with a pseudo-color bar (**b**). Scale bars are 50 μm. [Ca^2+^]_i_ dynamics in cells expressing empty vector pcDNA3.2 (**c**) or TRPV4 (**d**) from experiments as in **a**. Data are mean ± s.d. (*n* = 60 cells; r.u., relative unit). Arrows indicate the time of administration of hypo-osmolarity solution. Peak increases in [Ca^2+^]_i_ were calculated from experiments similar to these in **c** and **d** (**e**). Data are mean ± s.d. (*n* = 3 or 4 independent experiments). **f**–**i**, hypo-osmolarity-gated OSCA2.1 and OSCA2.2 channel activities were blocked by La^3+^. The cells expressing empty vector pcDNA3.2, OSCA2.1, or OSCA2.2 were incubated in standard bath solution (300 mOsm; − hypo-osmo) for 30 min and then treated with the hypo-osmotic solution containing 0.5 mM LaCl_3_ (140 mOsm; + hypo-osmo), and [Ca^2+^]_i_ were analyzed by Fura-2 emission ratios (**f**). Scale bars are 50 μm. Dynamic analyses of [Ca^2+^]_i_ increases in cells expressing empty vector pcDNA3.2 (**g**), OSCA2.1 (**h**), or OSCA2.2 (**i**) from experiments as in **f**. Data are mean ± s.d. (*n* = 6 ROI). Source Data

**Extended Data Fig. 6:**
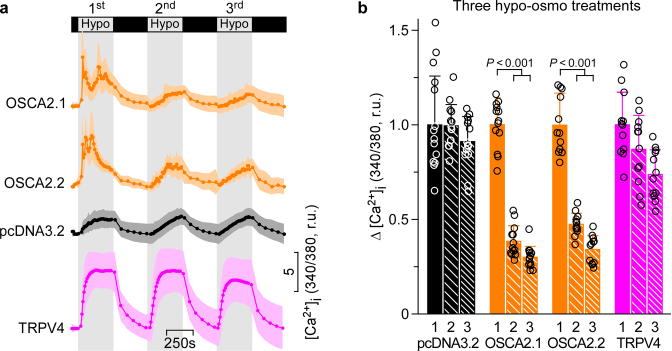
Desensitization of hypo-osmosensitive OSCA2.1 and OSCA2.2 Ca^2+^ channels expressed in HEK293 cells. **a**, Representative plots show HOSCA desensitization of OSCA2.1, OSCA2.2, and TRPV4 when expressed in HEK293 cells with pcDNA3.2 as a control. HEK293 cells were treated with the isosmotic solution (300 mOsm; black) and hypo-osmotic solution (140 mOsm; gray) repetitively three times, and [Ca^2+^]_i_ were analyzed by Fura-2 emission ratios. Data are mean ± s.d. (*n* = 3 independent experiments). Similar results were seen in > 10 times. **b**, Quantification of HOSCA desensitization from experiments similar to **a**. OSCA2.1 and OSCA2.2 showed over 50% of desensitization, while TRPV4 showed only about 10–20% of desensitization. The cells expressing pcDNA3.2 almost did not show any desensitization. The properties of HOSCA kinetics and desensitization for OSCA2.1 and OSCA2.2 expressed in HEK293 cells were consistent with the HOSCA kinetics *in planta* as shown in Fig. 3a,b. The 1^st^ HOSIC was arbitrarily set to 1, and data are mean ± s.d. (*n* = 3 or 4 independent experiments). Similar results were seen in > 10 times. Source Data

To determine whether OSCA2.1 and OSCA2.2 can facilitate Ca^2+^ influx in response to hypo-osmotic treatments, we analysed OSCA2.1 and OSCA2.2 in HEK293 cells^6,22^. In response to decreases in osmolarity, cells expressing OSCA2.1 or OSCA2.2 showed larger [Ca^2+^]_i_ increases than those harbouring empty vector (Fig. 3g–j). OSCA2.1–GFP and OSCA2.2–GFP were localized to the vicinity of the cell surface (images in Fig. 3i,j and Extended Data Fig. 5a). Similar to TRPV4, OSCA2.1 and OSCA2.2 enabled HOSCA in HEK293 cells, and their activities were blocked by La^3+^ (Fig. 3k and Extended Data Fig. 5b–i). HOSCA decayed similarly in cells expressing OSCA2.1 or OSCA2.2, and differed in cells expressing empty vector or TRPV4 (Fig. 3l). Similar to TRP desensitization^23,34,46^, in response to successive hypo-osmolarity challenges, robust desensitization was recorded in cells expressing OSCA2.1 or OSCA2.2 but not those with empty vector, whereas TRPV4-expressing cells displayed only mild desensitization (Extended Data Fig. 6). These results show that OSCA2.1 and OSCA2.2 have a distinct hypo-osmosensor activity.

## OSCAs control pollen Ca^2+^ oscillations

Pollen dehydration occurs before anthesis, which is vital for resistance to environmental stress during dispersal^9,10^. Although Ca^2+^ gradients and oscillations in pollen tubes were initially studied using microinjected Ca^2+^-sensitive dyes^41,43^, Ca^2+^ levels in pollen grains can only be detected using transgenic Ca^2+^ indicators^47,48^. Ca^2+^ signatures in pollen grains and their regulatory mechanisms as well as the causal relationship with germination thus remain poorly understood.

**Extended Data Fig. 7:**
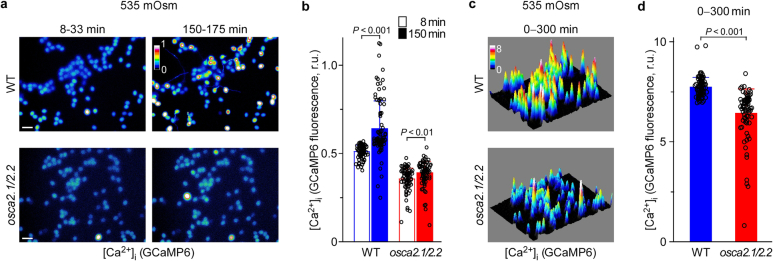
Ca^2+^ oscillations prior to germination in pollen grains placed on the standard media (535 mOsm) are impaired in the *osca2.1/2.2* double mutant. **a**,**b**, Representative cumulative Ca^2+^ images scaled by a pseudo-color bar at 8–33 min (left) and 150–175 min (right) are shown in pollen grains expressing GCaMP6 (**a**). Scale bars are 50 μm. Pollen grains from plants expressing GCaMP6 were placed on the common germination media (535 mOsm), and GCaMP6 fluorescence images were taken every 30 s for 300 min as shown in Fig. 4. Pollen GCaMP6 images for a total of 25 min at the indicated interval time points (8–33 min and 150–175 min) were pooled together, and scaled by a pseudo-color bar. The cumulative Ca^2+^ signals were analyzed using ImageJ and similar experiments were quantified (**b**; mean ± s.d.; *n*_WT_ = 91 grains, *n*_*osca2.1/2.2*_ = 82 grains). Similar results were seen in > 10 times. **c**,**d**, Cumulative pollen Ca^2+^ images for 300 min from experiments as in **a** and **b** were shown in 3D surface pseudo-color images (**c**) or bar graphs (**d**), respectively (mean ± s.d.; *n*_WT_ = 91 grains, *n*_*osca2.1/2.2*_ = 82 grains). Source Data

To analyse whether and how OSCA2.1 and OSCA2.2 act as hypo-osmosensors to control pollen Ca^2+^ signalling, we used GCaMP6m, which has high sensitivity and balanced kinetics^44^. We established a GCaMP6-based assay in wild-type pollen in standard medium, and observed [Ca^2+^]_i_ oscillations over a 300-min period (Supplementary Video 2). To take into consideration the variation of GCaMP6 expression and fluorescence bleaching associated with long recordings, we first analysed fluorescence intensities over a longer timescale to determine the broad trend in Ca^2+^ signalling during germination. Pollen [Ca^2+^]_i_ increased from the early stage of germination to about the germinating stage in both genotypes, but was weaker in *osca2.1/2.2* pollen, leading to lower total [Ca^2+^]_i_ over 300 min in *osca2.1/2.2* (Extended Data Fig. 7).

**Fig. 4:**
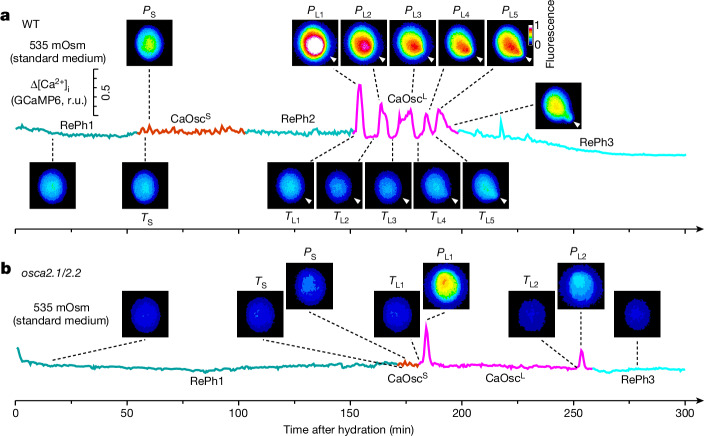
[Ca^2+^]_i_ oscillations in pollen grains prior to germination are impaired in *osca2.1/2.2.* **a**,**b**, Representative traces of GCaMP6 fluorescence recorded in a wild-type (**a**) or an *osca2.1/2.2* (**b**) pollen grain. Pollen expressing GCaMP6 were placed on standard germination medium and fluorescence images were taken every 30 s for 300 min. GCaMP6 images (scaled with a pseudocolor bar) at indicated time points are shown. The resting phases (RePh1, RePh2 and RePh3) were separated by [Ca^2+^]_i_ oscillations with small amplitudes (CaOsc^S^) or large amplitudes (CaOsc^L^). *P*_L_, peak CaOsc^L^ amplitude; *P*_S_, peak CaOsc^S^ amplitude; T_L_, trough CaOsc^L^ amplitude; T_S_, trough CaOsc^S^ amplitude. Arrowheads indicate the germination aperture. Vertical scale bar (panel **a**, top left): the whole scale is 0.5 r.u with 0.1 r.u. divisions. Similar results were seen more than 20 times. Source Data

To quantify systematically and statistically the timecourse of [Ca^2+^]_i_ oscillations, we recorded patterns of [Ca^2+^]_i_. We observed a first resting phase (RePh1), followed by [Ca^2+^]_i_ oscillations with small amplitudes (CaOsc^S^), then a second resting phase (RePh2), followed by [Ca^2+^]_i_ oscillations with large amplitudes (CaOsc^L^) that led to tube protrusion, and finally a third resting phase (RePh3) (Fig. 4a and Supplementary Video 2). Dozens of CaOsc^S^ appeared evenly distributed in the cytosol, and several CaOsc^L^ gradually propagated towards the germination aperture and finally triggered tube protrusion.

Typical *osca2.1/2.2* pollen had markedly reduced [Ca^2+^]_i_ oscillations, including but not limited to largely extended RePh1, shortened CaOsc^S^, abolished RePh2 and diminished CaOsc^L^ with fewer irregular spikes, which often did not trigger germination within 300 min (Fig. 4b). If pollen did not germinate, several CaOsc^S^ → RePh2 → CaOsc^L^ → RePh3-like modules re-occurred afterwards until germination was triggered in both genotypes, but much more so in *osca2.1/2.2* pollen. Given that OSCA2.1 and OSCA2.2 are hypo-osmosensors, the difference in Ca^2+^ spiking in *osca2.1/2.2* pollen suggests that Ca^2+^ spiking may function as a second messenger for extracellular water status.

## OSCAs couple external osmolarity to Ca^2+^ spiking

**Fig. 5:**
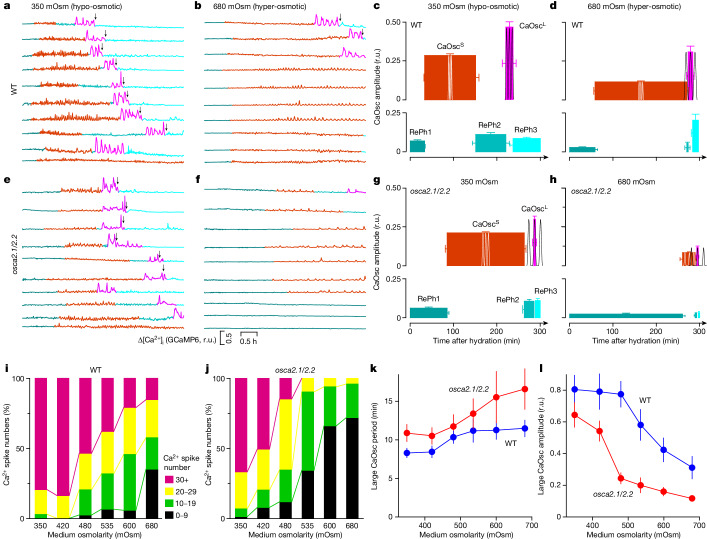
Induction of pollen [Ca^2+^]_i_ oscillations by gradual increases in water availability that mimic the rehydration process is disrupted in *osca2.1/2.2* pollen grains*.* **a**–**d**, Representative traces of GCaMP6 fluorescence from ten wild-type pollen grains placed on hypo-osmotic (**a**) or hyper-osmotic (**b**) medium show Ca^2+^ spiking and germination initiation (arrows). Trace colours are as shown in Fig. 4. Results from three independent experiments. Amplitudes, durations, periods and frequencies of Ca^2+^ traces under hypo-osmotic (**c**; *n* = 114 grains) and hyper-osmotic (**d**; *n* = 122 grains) conditions are shown. Data are mean ± s.e.m. Three oscillations are superimposed to illustrate the periods for CaOsc^S^ and CaOsc^L^. Similar results were seen more than ten times. **e**–**h**, Representative traces of GCaMP6 fluorescence from *osca2.1/2.2* pollen grains placed on hypo-osmotic (**e**) or hyper-osmotic (**f**) media. Ca^2+^ patterns under hypo-osmotic (**g**; *n* = 235 grains) and hyper-osmotic (**h**; *n* = 198 grains) conditions were quantified. Data are mean ± s.e.m. Similar results were seen more than ten times. **i**,**j**, Effects of medium osmolarity on the number of Ca^2+^ spikes. Wild-type and *osca2.1/2.2* pollen grains were placed on germination media with different osmolarities, and the numbers of CaOsc^S^ and CaOsc^L^ spikes were counted and grouped. Data from at least 6 replicates (*n* = 5 independent experiments). **k**,**l**, Effects of medium osmolarity on period (**k**) and amplitude (**l**) of CaOsc^L^ from the same experiments as in **i**,**j**. Data are mean ± s.d.; *n* = 5 independent experiments; two-way ANOVA, *P* < 0.001. Source Data

**Extended Data Fig. 8:**
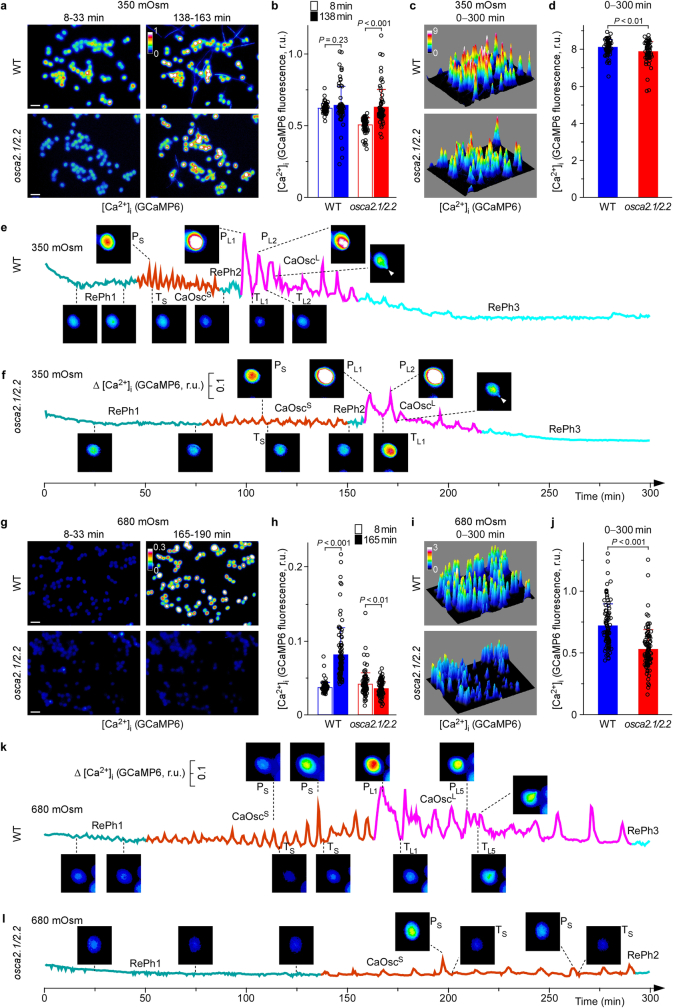
Pollen Ca^2+^-oscillation phenotype prior to germination in the *osca2.1/2.2* double mutant is rescued under the hypo-osmotic media (350 mOsm) and exacerbated under hyper-osmotic media (680 mOsm). **a**,**b**, Cumulative GCaMP6-fluorescence images were pooled together at the intervals of 8–33 min (left) and 138–163 min (right) for a total of 25 min, respectively, after pollen grains were spread on the hypo-osmotic (350 mOsm) germination media from WT and *osca2.1/2.2* plants (**a**), and quantified (**b**). Scale bars are 50 μm. GCaMP6-fluorescence images were collected every 30 s for 300 min, and are scaled by a pseudo-color bar. Data are mean ± s.d. (*n*_WT_ = 69 grains, *n*_*osca2.1/2.2*_ = 72 grains). Under the hypo-osmotic media WT pollen grains displayed an accelerated hydration, and at the time of 8–33 min the [Ca^2+^]_i_ were already increased to the level of these at the time of 138–163 min (as a saturated status), as shown in **a** and **b**. However, *osca2.1/2.2* pollen grains displayed a “normal” hydration as seen for the WT at the standard 535 mOsm media (Fig. 4a), and at the time of 8–33 min the [Ca^2+^]_i_ were lower, and then increased to the level at the time of 138–163 min. **c**,**d**, Cumulative pollen Ca^2+^ images for 300 min from the same experiments as in **a** and **b** were shown in 3D surface pseudo-color images (**c**) or bar graphs (**d**), respectively. Representative 3D surface plots of cumulative Ca^2+^ signals for 300 min after pollen grains were spread on the 350 mOsm germination media from WT and *osca2.1/2.2* plants. Data are mean ± s.d. (*n*_WT_ = 69 grains, *n*_*osca2.1/2.2*_ = 72 grains). Similar results were seen in > 10 times. **e**,**f**, Representative Ca^2+^ oscillations in a WT (**e**) or an *osca2.1/2.2* (**b**) pollen grain expressing GCaMP6 on the hypo-osmotic germination media (350 mOsm). GCaMP6-fluorescence images were taken every 30 s for 300 min. The time-lapse acquisitions show the peaks, troughs and resting phases of Ca^2+^ oscillations. Similar to the patterns Ca^2+^ oscillations in WT pollen grains in Fig. 4a, the resting phases (RePh_1_, RePh_2_ and RePh_3_) were separated with Ca^2+^ oscillations with small amplitude (CaOsc^S^) and large amplitude (CaOsc^L^) in both WT and *osca2.1/2.2* pollen grains. P_L_/P_S_, peak amplitude of CaOsc^L^/CaOsc^S^; T_L_/T_S_, trough amplitude of CaOsc^L^/CaOsc^S^. Similar results were seen in > 10 times. **g**,**h**, Cumulative GCaMP6-fluorescence images were pooled together at the intervals of 8–33 min (left) and 165–190 min (right) for a total of 25 min, respectively, after pollen grains were spread on the hyper-osmotic germination media (680 mOsm) from WT and *osca2.1/2.2* plants (**g**), and quantified (**h**). Scale bars are 50 μm. GCaMP6-fluorescence images were collected every 30 s for 300 min, and analyzed (mean ± s.d.; *n*_WT_ = 91 grains, *n*_*osca2.1/2.2*_ = 100 grains). **i**,**j**, Cumulative pollen Ca^2+^ images for 300 min from the same experiments as in **g** and **h** were shown in 3D surface pseudo-color images (**i**) or bar graphs (**j**), respectively. Data are mean ± s.d. (*n*_WT_ = 91 grains, *n*_*osca2.1/2.2*_ = 100 grains). **k**,**l**, Representative Ca^2+^ oscillations in a WT (**k**) or an *osca2.1/2.2* (**l**) pollen grain expressing GCaMP6 on the hyper-osmotic germination media (680 mOsm). GCaMP6-fluorescence images were taken every 30 s for 300 min. Largely altered patterns Ca^2+^ oscillations especially for *osca2.1/2.2* pollen grains feature extended the resting phase (RePh_1_) for *osca2.1/2.2* pollen grains and Ca^2+^ oscillations with small amplitude (CaOsc^S^) for WT pollen grains. P_L_/P_S_, peak amplitude of CaOsc^L^/CaOsc^S^; T_L_/T_S_, trough amplitude of CaOsc^L^/CaOsc^S^. Similar results were seen in > 10 times. Source Data

To further assess whether [Ca^2+^]_i_ oscillates faithfully according to water availability in the medium, we used hypo-osmotic medium, which resulted in *osca2.1/2.2* germination defects being largely restored, and hyper-osmotic medium, which resulted in prominent *osca2.1/2.2* germination defects (Fig. 2g). At 350 mOsm, the whole set of [Ca^2+^]_i_ signatures seen in Fig. 4a occurred in wild-type pollen, whereas at 680 mOsm, CaOsc^S^ were overwhelmingly extended over RePh2, CaOsc^L^ and RePh3 (Fig. 5a,b, Extended Data Fig. 8 and Supplementary Videos 3 and 4). [Ca^2+^]_i_ patterns were substantially altered under the hyper-osmotic condition, including but not limited to extended CaOsc^S^, delayed and/or diminished CaOsc^L^, and reduced amplitudes of CaOsc^S^ and CaOsc^L^ (Fig. 5c,d).

**Extended Data Fig. 9:**
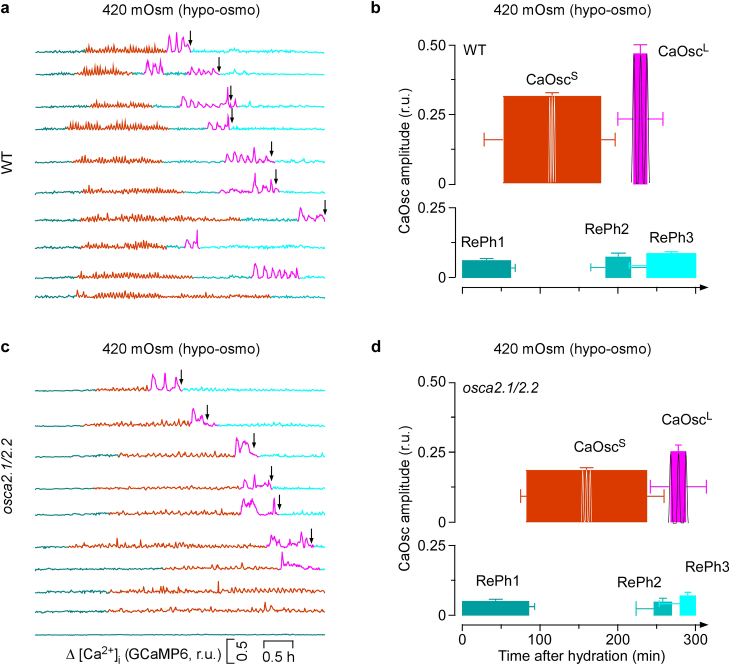
Restoration of pollen Ca^2+^-oscillation defects in *osca2.1/2.2* germination under the hypo-osmotic media (420 mOsm). **a**,**b**, Example traces of GCaMP6 fluorescence from 10 representative WT pollen grains placed on hypo-osmotic media (420 mOsm; **a**) showed Ca^2+^ spiking and germination initiation (arrows). The colors and their representations were the same as in Fig. 4 and Fig. 5a,b. Results from three independent experiments were shown. The amplitudes, durations, periods, and frequencies of these Ca^2+^ traces were quantified (**b**; mean ± s.e.m., *n* = 112 grains). These oscillations were superimposed to illustrate the properties for CaOsc^S^ and CaOsc^L^, as shown in Fig. 5c,d. Similar results were seen in > 5 times. **c**,**d**, Example traces of GCaMP6 fluorescence from 10 representative *osca2.1/2.2* pollen grains placed on hypo-osmotic media (420 mOsm; **c**) showed Ca^2+^ spiking and germination initiation, as seed in Fig. 5e,f. The Ca^2+^ spiking patterns under the hypo-osmotic condition were quantified (**d**; mean ± s.e.m., *n* = 118 grains). These oscillations were superimposed to illustrate the properties for CaOsc^S^ and CaOsc^L^, as seen in Fig. 5g,h. Similar results were seen in > 5 times. Source Data

**Extended Data Fig. 10:**
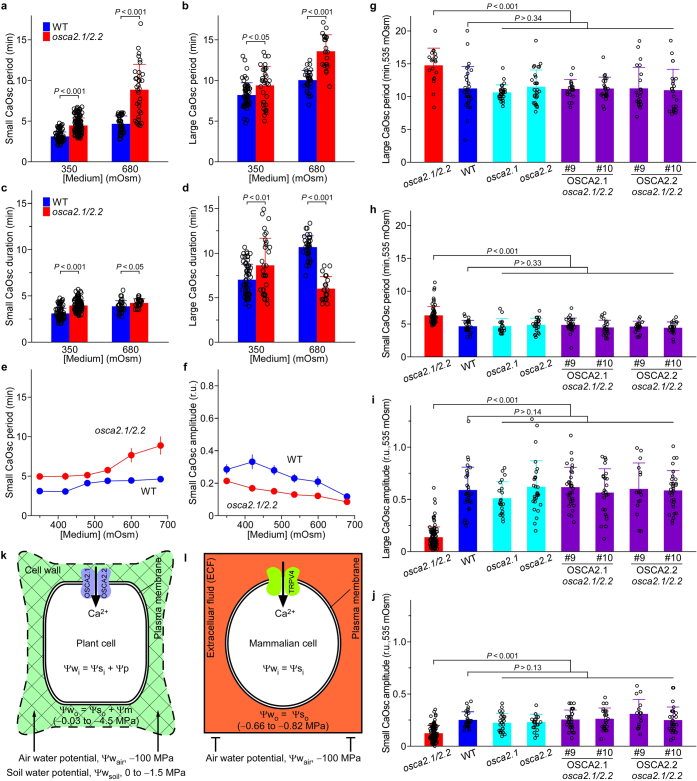
Impaired osmolarity regulation of [Ca^2+^]_i_ oscillations during pollen germination in *osca2.1/2.2.* **a**,**b**, Data on Ca^2+^ spiking patterns in pollen grains placed on hypo-osmotic (350 mOsm) or hyper-osmotic media (680 mOsm) were from the same experiments in Fig. 5a,b,e,f, and quantified for the periods of CaOsc^S^ (**a**) and CaOsc^L^ (**b**) similarly as in Fig. 5c,d,g,h (mean ± s.d.; *n*_350_ = 114 grains for WT; *n*_350_ = 235 grains for *osca2.1/2.2*; *n*_680_ = 122 grains for WT; *n*_680_ = 198 grains for *osca2.1/2.2*). The time between the peaks of two Ca^2+^ spicks was calculated as a period. The oscillation periods of CaOsc^S^ and CaOsc^L^ were increased under high osmolarity media in WT, suggesting that low water availability suppressed Ca^2+^ oscillations. Overall, the oscillation periods of CaOsc^S^ and CaOsc^L^ were longer in *osca2.2/2.2* than those in WT, suggesting that *osca2.2/2.2* had weaker Ca^2+^ oscillations. **c**,**d**, Data on Ca^2+^ spiking patterns in pollen grains placed on hypo-osmotic media (350 mOsm) or hyper-osmotic media (680 mOsm) were from the same experiments in Fig. 5a,b,e,f, and quantified for the oscillation duration times of CaOsc^S^ (**c**) and CaOsc^L^ (**d**) similarly as in Fig. 5c,d,g,h (mean ± s.d.; *n*_350_ = 114 grains for WT; *n*_350_ = 235 grains for *osca2.1/2.2*; *n*_680_ = 122 grains for WT; *n*_680_ = 198 grains for *osca2.1/2.2*). The duration time of an individual Ca^2+^ spick was calculated as the time between the troughs immediately before and after the peak. The duration times of CaOsc^S^ and CaOsc^L^ were increased under high osmolarity media in WT, also indicating that the water availability was positively associated with Ca^2+^ oscillations. Overall, the duration times of CaOsc^S^ and CaOsc^L^ were longer in *osca2.2/2.2* than those in WT, except for CaOsc^L^ at high osmolarity media. Note that, the shorter duration times of CaOsc^L^ at 680 mOsm in *osca2.2/2.2* were largely due to very few or no CaOsc^L^ occurring as seen in Fig. 5f, preventing accurate analysis. These results suggest that OSCA2.1/2.2 are responsible for sensing water, and that Ca^2+^ oscillations are the second messengers for water. **e**,**f**, Effects of media osmolarity on the period (**e**) and amplitude (**f**) of CaOsc^S^ from the same experiments as in Fig. 5i,j (mean ± s.d.; *n* = 5 independent experiments; two-way ANOVA, *P* < 0.001). With the decreases in media osmolarity, i.e. increases in water availability, the oscillation amplitudes were elevated and the oscillation periods were reduced, suggesting that the water availability was positively associated with Ca^2+^ oscillations. Overall, the *osca2.2/2.2* was less sensitive to the increases in water availability, consistent with OSCA2.1/2.2 being hypo-osmosensors. **g**–**j**, Complementation of defective Ca^2+^ oscillations in *osca2.1/2.2* under the standard germination media (535 mOsm) by expression of genomic DNA of OSCA2.1 or OSCA2.2. Data on Ca^2+^ spiking patterns in pollen grains placed on the 535 mOsm media were from the experiments similar to these in Fig. 4, and the periods and amplitudes of CaOsc^S^ (**g**, **h**) and CaOsc^L^ (**i**, **j**) were quantified similarly as in **a**–**d** (mean ± s.d.; *n* = 3 independent experiments). The periods and amplitudes of CaOsc^S^ and CaOsc^L^ in the *osca2.1* and *osca2.1* single mutants were similar to these in WT, consistent with their phenotypes in pollen germination (Fig. 2c) and HOSCA (Extended Data Fig. 4c–e). In addition, these periods and amplitudes of CaOsc^S^ and CaOsc^L^ in the complementation lines (*OSCA2.1 osca2.1/2.2* and *OSCA2.2 osca2.1/2.2*) were comparable to these in WT, also suggesting that OSCA2.1 and OSCA2.2 had functional redundancy in regulating Ca^2+^ oscillations. Notably and consistently, the periods of CaOsc^S^ and CaOsc^L^ in *osca2.1/2.2* were significantly longer than these of the rest of genotypes, and the amplitudes of CaOsc^S^ and CaOsc^L^ in *osca2.1/2.2* were also significantly smaller than these of the rest of genotypes. Taken together, these WT-like phenotypes on Ca^2+^ oscillations, germination, and HOSCA in the *osca2.1* and *osca2.1* single mutants and complementation lines suggest that OSCA2.1 and OSCA2.2 were redundant and function together to control pollen grain perception of hypo-osmolarity conditions. **k**, Model of osmosensors and water potential relationships in plant cells and their environment. Soil water potential (Ψw_soil_) drops from saturated state (0 MPa) to field capacity (−0.03 MPa), at which both air and water are in the macropores, to permanent wilting point (−1.5 MPa), at which soil water is held by solid particles too tightly to be taken up by plants^7,85,87^. The air water potential is about −100 MPa, which provides the driving force for water transport through the soil−plant-atmosphere continuum. Notably, the extracellular spaces (called the apoplast) are connected largely with their environments (illustrated by a dashed line surrounding cell walls and arrows). The extracellular spaces in roots are directly connected to the soil, and stomatal pores allow the extracellular spaces in the leaf to connect to the air, forming the soil-plant-atmosphere continuum. Therefore, the water potential in the extracellular spaces (outside of the cell, Ψw_o_) is not set to a point, in contrast to that in mammalian cells, and varies enormously from −0.03 to −4.5 MPa (equivalent to the change in solution osmolarity from 12 to 1820 mOsm). As the osmosensing OSCA1 family has expended greatly compared to three members in animals, and co-evolved with the plant transition from water to land^35^, these OSCAs may be major osmosensors in plants. **l**, Model of osmosensors and water potential relationships in mammalian cells and their environment. The extracellular spaces are separated with their environment (illustrated by a solid line and stop arrows). The water potential of the extracellular fluid (ECF, Ψw_o_) is set to a point, and the ECF osmolarity is tightly regulated around this set-point of ∼ 300 ± 30 mOsm (equivalent to the change in water potential from −0.66 to −0.82 MPa)^12^. Source Data

In 350 mOsm medium, [Ca^2+^]_i_ patterns in *osca2.1/2.2* pollen resembled those seen in the wild-type pollen at 535 mOsm, however, [Ca^2+^]_i_ spiking was almost abolished at 680 mOsm (Fig. 5e–h). The CaOsc^S^ and CaOsc^L^ had reduced amplitudes and delayed initiation times in *osca2.1/2.2* pollen. Results similar to these at 350 mOsm were also observed in the 420 mOsm hypo-osmotic medium (Extended Data Fig. 9), consistent with the germination phenotypes. For comparison, we quantified the period and the duration of individual spikes, and found that CaOsc^S^ and CaOsc^L^ in *osca2.1/2.2* pollen had longer periods and longer durations (Extended Data Fig. 10a–d). Together, these results show that Ca^2+^ spiking in pollen was tightly controlled by the osmolarity of the medium, and that this link was weakened in *osca2.1/2.2* pollen. Of note, prolonged pollen Ca^2+^ spiking and intensified Ca^2+^ spiking at the germination aperture have been observed previously, but were not quantified^48^.

We assessed the number of [Ca^2+^]_i_ spikes in pollen placed on media with osmolarity reduction from 680 mOsm to 350 mOsm to mimic increases in water availability during rehydration (Fig. 2g). Decreases in osmolarity increased the total number of spikes in both genotypes, but much less in *osca2.1/2.2* (Fig. 5i,j). The periods of CaOsc^S^ and CaOsc^L^ were shortened and their amplitudes were increased in an osmolarity-dependent manner (Fig. 5k,l and Extended Data Fig. 10e,f), illustrating that Ca^2+^ spiking was enhanced during rehydration, whereas *osca2.1/2.2* pollen exhibited much weaker Ca^2+^ spiking. To further verify the causal relationship between [Ca^2+^]_i_ oscillations and OSCA2.1 and OSCA2.2, we analysed *osca2.1*, *osca2.2* and *osca2.1/2.2* complementation lines. [Ca^2+^]_i_ oscillations were not affected in *osca2.1* or *osca2.2* pollen, and OSCA2.1 and OSCA2.2 rescued *osca2.1/2.2* defects in respect to the periods and amplitudes of CaOsc^S^ and CaOsc^L^ (Extended Data Fig. 10g–j). These findings demonstrate that the coupling of medium osmolarity to [Ca^2+^]_i_ oscillations is mediated by OSCA2.1 and OSCA2.2, serving as a molecular mechanism for sensing water availability during rehydration.

## Discussion

Here we answer two long-standing questions regarding the nature of the molecular nature of HOSCA and whether HOSCA functions as a hypo-osmosensing mechanism. We also reveal that Ca^2+^ oscillations serve as a second messenger for a primary stimulus in pollen grains. Since second messengers are intracellular small molecules that transfer the extracellular signal received by cell surface receptors to the cytosol, our results reveal that OSCA2.1 and OSCA2.2 perceive extracellular osmolarity and convert to Ca^2+^ spiking in pollen. Note that although the exact gating mechanisms remain unknown, OSCA family members may be hypo- or hyper-osmosensitive, similar to TRP family members sensing hot and cold temperatures^23,34,46^.

A central question is whether Ca^2+^ spiking could serve specifically as a second messenger for osmolarity in extracellular spaces, even though other cellular processes that occur during rehydration are also induced by water. Here we present several lines of evidence that this is likely. *osca2.1/2.2* pollen did not germinate in normal 535 mOsm medium, suggesting that the initiation switch controlled by OSCA2.1 and OSCA2.2 is required even when there is sufficient water for germination. *osca2.1/2.2* pollen also did not develop normal Ca^2+^ oscillations at 535 mOsm, suggesting that OSCA2.1 and OSCA2.2 are required for these signals. In addition, *osca2.1/2.2* pollen did not display HOSCA, indicating that OSCA2.1 and OSCA2.2 sense hypo-osmotic shock. OSCA2.1 and OSCA2.2 also formed Ca^2+^-permeable channels, indicating that they may directly convert hypo-osmotic stimulus into Ca^2+^ increases. Together, these results closely link Ca^2+^ spiking to medium osmolarity via OSCA2.1 and OSCA2.2, establishing that water in the medium, OSCA2.1 and OSCA2.2, and Ca^2+^ spiking form a novel sequential signalling cascade.

The water potential (*Ψ*_w_) controls water movements across cell membranes^7,8,12,14^. *Ψ*_w_ is composed of solute potential (*Ψ*_s_), hydrostatic pressure potential (*Ψ*_p_) and matrix potential (*Ψ*_m_) (Extended Data Fig. 10k). In freshwater land plants, *Ψ*_w_ in the extracellular solution (${\psi }_{{{\rm{w}}}_{o}}$) varies from −0.03 to −4.5 MPa (equivalent to a 150-fold difference in osmolarity from 12 to 1,800 mOsm). By contrast, in mammals there is a limited range of osmolarity fluctuations from −0.66 to −0.82 MPa, about 10% around the set-point of approximately 300 mOsm (Extended Data Fig. 10l). The large detection ranges of OSCA2.1 and OSCA2.2 in plants compared with hypo-osmosensors in animals is likely to be owing to the presence of a cell wall in plants. Desiccated seeds and pollen grains^7,8^ can have extremely low *Ψ*_w_ of less than −100 MPa. During rehydration, ${\psi }_{{{\rm{w}}}_{o}}$ increases gradually, and when the cell membrane is established, ${\psi }_{{{\rm{w}}}_{o}}$ exceeds the *Ψ*_w_ in the cytosol (${\psi }_{{{\rm{w}}}_{i}}$), which activates hypo-osmosensors, leading to Ca^2+^ influx.

Ca^2+^ spiking with distinct signatures occurs in various signalling processes in plants, including nodulation and mycorrhizal symbiotic establishment in root hairs, abscisic acid-induced stomatal closure, circadian oscillations, diatom osmoregulation and pollen tube tip growth^9,19,21,41^. Ca^2+^ spiking in pollen grains resembles Ca^2+^ oscillations in pollen tube tips^41,48^, although pollen Ca^2+^ spiking occurred less evenly and with greater fluctuations in amplitudes and periods, and with periods longer than those in pollen tubes^43^ (4–10 min versus around 20 s). Whether OSCA2.1 and OSCA2.2 have a role in Ca^2+^ oscillations in pollen tubes remains to be determined. Notably, several Ca^2+^ channels have been associated with Ca^2+^ oscillations in pollen tube tips, but the gating mechanisms and roles remain poorly understood^9,10^. For instance, cyclic nucleotide-gated channels and glutamate-like receptors are known to be involved in Ca^2+^ oscillations in pollen tubes^9,49,50^. In addition, cell surface MSL8 permeates anions and regulates pollen volume and integrity but inhibits pollen germination^45^. A key open question is how OSCA2.1 and OSCA2.2 work in concert with such channels to monitor water availability to generate Ca^2+^ spikes essential for pollen germination, tube growth and egg fertilization. It is possible that at a given osmolarity, many osmosensors, including hyper- and hypo-osmosensors, might function together to maintain general Ca^2+^ homeostasis in pollen. Our identification of the water → OSCA2.1–OSCA2.2 → Ca^2+^ spiking pathway in pollen grains will open new avenues for dissecting Ca^2+^ oscillations in pollen tubes. Nevertheless, although water is the most important driving force for cell growth^3,7,14,43^, it remains to be determined whether this water–hypo-osmosensor–Ca^2+^ spiking pathway exists in vegetative tissues. The OSCA family originated during the evolution of protists^35^, implying a role in maintaining cell shape and turgor^5,6^. Moreover, plant OSCAs expended greatly and evolved coincidently with the plant transition from water to land^35^, suggesting an essential role in plant responses to water fluctuations. In conclusion, we have identified and characterized OSCA2.1 and OSCA2.2 as the long-sought hypo-osmosensitive Ca^2+^ channels in plants, and identified the role of Ca^2+^ spiking as the second messenger for water availability during pollen germination.

## Methods

### Screen based on functional expression of OSCA in *E. coli*

The bacterial growth assay was designed with the consideration of either the complementary effect or the toxicity effect caused by ion channels as described^39,40,51–53^. DNA sequences encoding 15 *Arabidopsis* OSCA family members^6^ were cloned into the Gateway entry plasmid pENTR/D-TOPO, and then into the destination plasmid pDEST14 for T7 promoter-based expression. Normal lysogeny broth (LB) medium contains 1% tryptone, 0.5% yeast extract and 171 mM NaCl (417 mOsm). Low-salt hypo-osmotic lysogeny broth (LSHypo-LB) medium was modified from LB medium, and contained 1% tryptone, 0.5% yeast extract, 5 mM NaCl and 3 mM KCl (70 mOsm). The osmolarity of LSHypo-LB medium was further adjusted by adding sorbitol as indicated. For spot growth assays, OSCA plasmids were transferred into the BL21(DE3) pLysS strain, which provides a tight control for expression of toxic proteins, and the freshly transformed strains were spread on an ampicillin and chloramphenicol plate and incubated overnight at 37 °C. Cultures were centrifuged, and precipitations were re-suspended using the LSHypo-LB medium. Cells were adjusted to optical density at 600 nm (OD_600_) of 0.2, and serially diluted at 10×, 10^3^× and 10^5^×. These dilutions were spotted at the volume of 1.5 μl per spot onto LSHypo-LB plates. IPTG (1 mM), sorbitol, LaCl_3_ and GdCl_3_ at the indicated concentrations were added. Bacterial spot cultures were photographed and the bacterial growth rate was analysed using ImageJ^54^. Osmolarity was measured with a vapour pressure osmometer (VAPRO 5600, Wescor).

### Plant materials and growth conditions

*Arabidopsis thaliana* (Col-0) was used as the wild type. *A. thaliana* (Col-0) stably expressing GCaMP6m under the control of pollen-specific *LAT52* promoter were generated and also used as the wild-type. The *osca1.1-1* mutant was from our previous study^6^. *Arabidopsis* T-DNA insertion lines (Col-0) for 15 OSCAs were obtained from the ABRC and the GABI-Kat project (Supplementary Table 1), and the homozygous T-DNA insertion lines were verified as described previously^6^. *Arabidopsis* plants were grown on soil (Sungro, Professional growing mix), or in Petri dishes in half-strength Murashige and Skoog salts (0.5× MS; Sigma), 1% (w/v) sucrose (Sigma), and 0.8% (w/v) agarose (Sigma) in controlled environmental chambers or rooms at 21 ± 2°C and 65% relative humidity. The fluency rate of white light was ~110 μmol m^−2^ s^−1^. The photoperiods were 16 h light/8 h dark cycles. *Arabidopsis* seeds were sown on soil or 0.5× MS medium, placed at 4 °C for 4 days in the dark, and then transferred to growth rooms.

### In vitro pollen germination assay

Pollen grains from freshly opened mature flowers (stage 12–13) of 8–12 week-old *Arabidopsis* plants were dispersed onto standard solid pollen germination medium containing 0.5% agarose, 300 mM (9.7% w/v) sucrose, 225 mM sorbitol, 1.6 mM boric acid, 1 mM CaCl_2_, 1 mM Ca(NO_3_)_2_ and 1 mM MgSO_4_ (adjusted to pH 6.3 with KOH; adjusted to osmolarity 535 mOsm with sorbitol), and placed at room temperature (22–24 °C) for 6 h for the in vitro germination assay^55–61^. Pollen grains were photographed using inverted fluorescence microscopes (Axio Observer 3; Zeiss) equipped cooled CCD/CMOS cameras (CoolSNAP HQ2/Prime 95B; Teledyne Photometrics)^6,62^ and MetaMorph 7.7 and MetaFluor 7.7 (Molecular Devices), and the germination rate was analysed using ImageJ. Osmolarity was measured with a Wescor vapour pressure osmometer.

### In vivo pollen germination assays

In vivo pollen germination analyses were carried out by two approaches: imaging pollen constitutively expressing GFP driven by a pollen-specific *LAT52* promoter^58,63^, and aniline blue staining^64,65^. The *osca2.1/2.2* mutant was crossed into wild-type *Arabidopsis* expressing *pLAT52*-driven GFP (*pLAT52::GFP*) and homozygous lines were obtained. Flowers from the wild-type plant (not expressing GFP) were emasculated 24 h prior to pollination and left in the growth chamber or room until in vivo pollen germination assay. Pollen grains with or without *pLAT52*-driven GFP expression from newly opened flowers were dabbed onto the surfaces of pre-emasculated wild-type stigmas. For GFP-visualization assay, after 30 min of pollination, stigmas with *pLAT52*-driven GFP-expressing pollen grains were removed from the plants, placed on the cover glass, and imaged using a fluorescence stereo microscope (Axio Zoom.V16; Zeiss) equipped with a CCD camera (Axiocam MR R3, Zeiss). Excitation was provided at 488 (20) nm, and GFP fluorescence images at 509 (10) nm emission were collected using Zen 2012 software (Zeiss). For aniline blue staining, after 2 h of pollination using pollen grains without GFP expression, pistils were excised and fixed in Carnoy’s fixative (75% ethanol and 25% acetic acid), softened in 10 M NaOH, and stained in 0.1% aniline blue^64,65^. Stained pistils were observed under the Axio V16 microscope, and fluorescence images were collected with excitation at 359 (20) nm and emission at 457 (20) nm. Because non-germinated grains were washed out for the aniline blue staining, the number of germinated grains that adhered to the stigma were counted. These in vivo germination analyses for wild type and *osca2.1/2.2* were carried out side-by-side and with switched order between these genotypes to minimize variations, and the data represent more than five independent experiments.

### Imaging of [Ca^2+^]_i_ in HEK293 cells

Analyses of ion channel activities in HEK293 cells were carried out as described previously^6,62,66–68^. HEK293 cells were cultured and maintained in DMEM supplemented with 10% fetal bovine serum, 1% penicillin and streptomycin in CO_2_ incubators at 37°C. For transfection, cells were seeded onto poly-lysine-coated eight-well chambered cover glasses (Nunc) overnight and transfected with plasmid DNA using Lipofectamine 3000 (Invitrogen). Cells were loaded with Fura-2AM (5 μM; Sigma), and a Fura-2-based Ca^2+^ imaging assay was performed in cells 18 to 24 h after transfection using an inverted fluorescence microscope (Axio Observer 3) equipped with two filter wheels (Lambda 10-3; Sutter Instruments), and cooled CCD and CMOS cameras (CoolSNAP HQ2/Prime 95B; Teledyne Photometrics)^6,62^. Emission ratiometric images (*F*_340 nm_/*F*_380 nm_) were collected using MetaFluor software or Micro-Manger software (https://micro-manager.org/). Cells were incubated in a standard buffer containing 50 mM NaCl, 3 mM KCl, 0.6 mM MgCl_2_, 10 mM glucose, 0.1 mM CaCl_2_, 10 mM HEPES, and 160 mM mannitol (adjusted to pH7.4 with NaOH and osmolarity 300 mOsm with mannitol) for 30 min. For hypo-osmotic treatment, the bath was perfused using a peristaltic pump (Dynamax RP-1; Rainin) with hypotonic solution (140 mOsm; standard buffer without 160 mM mannitol), and HOSCA were recorded. For HOSCA desensitization analysis, cells were incubated in standard isosmotic solution (300 mOsm), and then treated with standard hypo-osmotic solution (140 mOsm) 3 times as illustrated in Extended Data Fig. 6a. Osmolarity was measured with a Wescor vapour pressure osmometer.

### Analysis of OSCA–GFP/YFP expression

For analysis of OSCA–GFP in HEK293 cells^6^, cells were transfected transiently with *pCMV::OSCA2.1-GFP*, *pCMV::OSCA2.2-GFP* or *pCMV::GFP*, and about 18 to 24 h after transfection, GFP fluorescence imaging was carried out using a confocal microscope (LSM710 or LSM880 with Airyscan; Zeiss). For analysis of OSCA–GFP or OSCA–YFP in *Arabidopsis*, both OSCA endogenous promoter OSCA–YFP and *pLAT52-*driven OSCA–GFP transgenic single-insertion homozygous lines (*pOSCA2.1::OSCA2.1-YFP*, *pOSCA2.2::OSCA2.2-YFP*, *pLAT52::OSCA2.1-GFP* and *pLAT52::OSCA2.2-GFP*) were generated as described^6,25,61,69,70^. The *pLAT52::GFP* transgenic plants were used as controls. Whole seedlings were imaged with the stereo microscope (Axio Zoom.V16) or the confocal microscope. The fluorescence in pollen grains and pollen tubes was analysed using a LSM880 confocal microscope with Airyscan. The plasma membrane was labelled with the FM4-64 dye and analysed as described previously^43,71–73^ using the confocal microscope. For each transgenic line, more than ten independent transformants were analysed and similar results were obtained. The plasma membrane localization is also consistent with the prediction by SUBA4 (http://suba.plantenergy.uwa.edu.au/)^74^.

### Histochemical GUS activity analysis

Histochemical staining for GUS activity using the OSCA endogenous promoter-driven OSCA full-length genomic DNA–GUS (*pOSCA2.1::*genomic*_OSCA2.1-GUS*, *pOSCA2.2::*genomic*_OSCA2.1-GUS*) transgenic lines as described^6,25^. Seedlings grown in 0.5× MS medium or soil were used for the histochemical staining^75^. Data represent more than five independent lines, which displayed similar staining patterns. Flowers and seedlings were placed into GUS reaction solution, and imaged microscopically (SteREO Discover V20, Zeiss).

### Pollen grain viability assay

Pollen grains from mature flowers at stage 12–13 were used for viability assay^45,76,77^. Pollen grains were released on slides containing the solid germination medium (420 mOsm), the slides were placed upside down and incubated for 30 min in a chamber with ∼ 95% relative humidity. Then 100-μl drops of solution containing 1 μg ml^−1^ fluorescein diacetate (FDA) and 0.5 μg ml^−1^ propidium iodide (PI) were added. FDA and PI fluorescence was recorded in the GFP and dsRED fluorescence channels, respectively, using the confocal microscopy. FDA stains live grains, while PI stains the edge of live grains as well as compromised grains.

### Imaging of [Ca^2+^]_i_ in pollen grains

Wild-type (Col-0) expressing GCaMP6 (ref. ^44^) driven by the pollen-specific *LAT52* promoter^48,49,72^ (*pLAT52::GCaMP6m*) was used as the wild type. The *osca2.1* and *osca2.2* single mutants and the *osca2.1/2.2* double mutant expressing GCaMP6, *osca2.1* GCaMP6, *osca2.2* GCaMP6 and *osca2.1/2.2* GCaMP6, respectively, were used as *osca2.1* and *osca2.2* and *osca2.1/2.2* mutants. *OSCA2.1 osca2.1/2.2* GCaMP6 and *OSCA2.2 osca2.1/2.2* GCaMP6 were generated using lines 9 and 10 described in Extended Data Fig. 3f, and used as complementation lines. Imaging and analysis [Ca^2+^]_i_ in pollen grains were carried out as described in previous studies with modifications^6,25,56,72,78–83^. Pollen grains from freshly opened mature (stage 12–13) flowers were dispersed on the solid germination medium with indicated osmolarity. For GCaMP6-based [Ca^2+^]_i_ imaging, excitation was provided at 485 nm, and 510 nm emission images were taken with an exposure time of 100 ms and collected at the indicated intervals using the Axio Observer 3 microscope. More than 50 grains were imaged in one view each time, and GCaMP6 fluorescence intensity for each individual pollen grain was obtained for further analysis. For the analysis of relative [Ca^2+^]_i_, GCaMP fluorescence Δ*F*/*F*_0_ was calculated as (*F* − *F*_0_)/*F*_0_, where *F*_0_ is the baseline fluorescence signal averaged over the first ten stable frames immediately before the start of treatments, and analysed using ImageJ.

For the hypo-osmotic shock treatment in plants, hyper-osmotic conditions were applied for a relatively long time (hours) first to generate a stable high osmotic status in plants, and then hypo-osmotic solutions were applied quickly for analysis of hypo-osmotic signalling processes that occur in minutes, which have been well described for various studies, including Ca^2+^ signalling^16–18,84^. For analysis of hypo-osmotic shock-induced cytosolic free Ca^2+^ concentration ([Ca^2+^]_i_) increases (HOSCA), pollen grains from freshly opened mature (stage 12–13) flowers were dispersed on high-osmolarity media modified from standard solid germination medium (535 mOsm) by adding sorbitol at the indicated concentrations for 1 h, and then perfused with the low-osmolarity solution modified from the standard solid germination solution without addition of sorbitol and agarose. Standard solid pollen germination medium contained 0.5% agarose, 300 mM (9.7% w/v) sucrose, 225 mM sorbitol, 1.6 mM boric acid, 1 mM CaCl_2_, 1 mM Ca(NO_3_)_2_, and 1 mM MgSO_4_ (adjusted to pH 6.3 with KOH; adjusted to osmolarity 535 mOsm with sorbitol). GCaMP florescence images were collected every 2 s with an exposure time of 100 ms for 350 s. Osmolarity was measured with a Wescor vapour pressure osmometer. Hypo-osmotic solution, which was prepared as the standard solution without the addition of 225 mM sorbitol, was added into the bath at the indicated time during continues imaging.

For the analysis of [Ca^2+^]_i_ oscillations in pollen grains, a two-step approach was used: visual analysis of the GCaMP6 fluorescence video, and quantification of the GCaMP6 fluorescence, both of which were then matched and adjusted for accuracy. First, the GCaMP6 fluorescence video, which was composed of images taken for pollen grains placed on germination medium every 30 s for 300 min, such as Supplementary Videos 2–4, was played using a 55 inch QLED 4 K TV monitor (Q80, Samsung) in a darkroom, and individual Ca^2+^-spiking events of [Ca^2+^]_i_ oscillations with small amplitudes (CaOsc^S^) and large amplitudes (CaOsc^L^) and their corresponding image number and time for each pollen gain were manually identified and counted. CaOsc^S^ were eye-detectable ‘flashes’ with not only increases in fluorescence intensity (about 2–3 times the baseline intensity), but also increases in the area of fluorescence. CaOsc^L^ were intensive flashes with increases in fluorescence intensity to 5 times or more the baseline intensity. Second, individual grains in the fluorescence images at the initial stage (taken within 5 min) were circled or drawn manually as regions of interest, and florescence intensities of regions of interest throughout the germination process for 300 min were calculated. Note that increases in pollen fluorescence area were not incorporated into the quantitative analyses. The fluorescence baseline was adjusted with fluorescence photobleaching correction. Oscillations of fluorescence intensity were matched to these CaOsc^S^ and CaOsc^L^ events, which were obtained by visual analysis in the first step, and classified into the simplest categorizations of relatively quiet resting phases (RePh1, RePh2 and RePh3) and CaOsc^S^ and CaOsc^L^ as described in detail in the main text.

### Water potential

*Ψ*_w_ is composed of *Ψ*_s_, *Ψ*_p_ and *Ψ*_m_ (interaction with matrices of solids and of macromolecules, such as cell walls) in plants^7^: *Ψ*_w_ = *Ψ*_s_ + *Ψ*_p_ + *Ψ*_m_. The osmotic potential of pure water is zero, and the osmotic potential for a solution is always negative. The osmotic potential of a solution (in molarity) is calculated by using the following formula: *Ψ*_s_ = −*iCRT*, where i is ionization constant, C is molar concentration, R is the pressure constant, and T is the absolute temperature. The water potentials in plant cells and mammalian cells illustrated in Extended Data Fig. 10k,l are largely estimated using information from the literature^7,8,12,85–87^—actual water potentials for a given species may vary greatly.

### DNA constructs and transgenic lines

For *E. coli* growth assay, cDNAs encoding 15 *Arabidopsis* OSCA family members^6^ were amplified by PCR using primers listed in Supplementary Table 2. The PCR products were cloned into the Gateway entry vector pENTR/D-TOPO vector in host cell TOP10, and the verified pENTR/D-TOPO vectors containing interesting gene were subsequently recombined into the destination vector pDEST14 for OSCA gene expression in *E. coli* BL21(DE3) pLysS cells.

The *osca2.1 osca2.2* double mutant (*osca2.1/2.2*) was generated by crossing *osca2.1* and *osca2.2* (Extended Data Fig. 3a,b). For complementation, clones containing *OSCA2.1* promoter driving *OSCA2.1* genomic DNA or *OSCA2.2* promoter driving *OSCA2.2* genomic DNA were amplified by PCR using primers (Supplementary Table 2). The transgenic lines of *osca2.1/2.2* expressing *pOSCA2.1::OSCA2.1* (*OSCA2.1 osca2.1/2.2*) or *pOSCA2.2::OSCA2.2* (*OSCA2.2 osca2.1/2.2*) were generated via floral dip transformation as described previously^6,69^.

For HEK293 Ca^2+^ imaging analysis, cDNAs encoding *Arabidopsis* OSCA2.1 and OSCA2.2, and mouse TRPV4 were amplified by PCR using primers (Supplementary Table 2). The PCR products were cloned into the Gateway entry vector and subsequently the destination vector pcDNA3.2 for gene expression in HEK293 cells.

For OSCA–GFP analysis in HEK293 cells, cDNAs encoding OSCA2.1 or OSCA2.2 were cloned into the pEGFP-N1 vector (Clontech), and these plasmids were used to transfect HEK293 cells.

For OSCA–GFP or OSCA–YFP analysis in *Arabidopsis*, clones containing *OSCA2.1* promoter driving *OSCA2.1* genomic DNA or *OSCA2.2* promoter driving *OSCA2.2* genomic DNA were amplified by PCR using primers listed in Supplementary Table 2. The promoter and the DNA fragments were fused by PCR and the products were cloned into the Gateway pENTR-1A vector, which were subsequently recombined into the destination vector pGWB540 to express GFP fusions with OSCA2.1 or OSCA2.2 in *Arabidopsis* via transformation^6,69^.

For GUS activity analysis, the genomic DNA containing the promoter and coding sequence for OSCA2.1 or OSCA2.2 were amplified by PCR using primers listed in Supplementary Table 2, cloned into the Gateway pENTR-1A vector, and subsequently recombined into the destination vector pGWB533 to express tissue-specific promoter-driven GUS in *Arabidopsis* via transformation^6,69^.

For imaging of [Ca^2+^]_i_ in pollen grains, wild-type (Col-0) expressing GFP fluorescence-based Ca^2+^ indicator GCaMP6m (GCaMP6)^44^ driven by the pollen-specific *LAT52* promoter^48,49,72^ (*pLAT52::GCaMP6m*) was generated by transformation^6,69^. The *osca2.1* and *osca2.2* single mutants and the *osca2.1/2.2* double mutant were crossed into the wild-type expressing GCaMP6, and over five independent homozygous lines of *osca2.1* GCaMP6, *osca2.2* GCaMP6, and *osca2.1/2.2* GCaMP6 were obtained and analysed with similar results. GCaMP6-expressing lines of *OSCA2.1 osca2.1/2.2* (*OSCA2.1 osca2.1/2.2* GCaMP6) and *OSCA2.2 osca2.1/2.2* (*OSCA2.2 osca2.1/2.2* GCaMP6) were generated accordingly by transformation^6,69^.

### OSCA mRNA analysis

*OSCA2.1* and *OSCA2.2* RNA were isolated from leaves using a RNA extraction kit and reverse transcription kit (Bioline). The abundance of mRNAs from the wild-type and *osca2.1/2.2* seedlings was analysed by RT–PCR, and *UBQ* was used as a loading control as described^6,88^. Gene-specific primers (Supplementary Table 2) were used for *OSCA2.1*, *OSCA2.2* and *UBQ* expansion by standard PCR.

### Statistics and reproducibility

To minimize the system variations, wild-type and *osca* mutants as well as transgenic lines were grown side-by-side in the agarose medium in Petri dishes or in soil in trays, and the Petri dishes and trays were rotated every other day in positions in the growth chambers or rooms to establish relatively even temperature and light. Independent experiments were performed at least three times with similar results, unless indicated otherwise. The statistical analysis was performed using EXCEL 2016 software (Microsoft), and *P* values were calculated via unpaired (two components with equal variance) or two-tailed *t*-test for bar graphs, unless stated otherwise. Data are presented as mean ± s.d. or mean ± s.e.m. To analyse the difference between genotypes in curve or line graphs, two-way ANOVA was carried out using SAS software (SAS Institute). Values of *P* < 0.05 were considered statistically significant.

### Reporting summary

Further information on research design is available in the Nature Portfolio Reporting Summary linked to this article.

## Online content

Any methods, additional references, Nature Portfolio reporting summaries, source data, extended data, supplementary information, acknowledgements, peer review information; details of author contributions and competing interests; and statements of data and code availability are available at 10.1038/s41586-024-07445-6.

### Supplementary information

Supplementary Information This file contains Supplementary Figure 1: Gel source data; Supplementary Table 1: OSCA T-DNA insertion lines and primers used for genotyping; and Supplementary Table 2: PCR primers and vectors.

Reporting Summary

Supplementary Video 1 Hypo-osmolarity-induced Ca^2+^ increases (HOSCA) are disrupted in *osca2.1/2.2* pollen grains. Pollen grains from wild type and *osca2.1/2.2* plants expressing the Ca^2+^ indicator GCaMP6 were hydrated in the germination medium containing 300 mM sorbitol for 1 h, and GCaMP6 fluorescence images were taken every 1 sec for 300 sec after treated with the germination solution containing 0 mM sorbitol. The same GCaMP6 images scaled by a pseudocolour bar were shown in Fig. 3a and quantified in Fig. 3b,c.

Supplementary Video 2 Pollen Ca^2+^ oscillations prior to germination are impaired in *osca2.1/2.2*. Pollen grains from wild type and *osca2.1/2.2* plants expressing the Ca^2+^ indicator GCaMP6 were placed on the standard germination medium (535 mOsm), and GFP fluorescence images were taken every 30 s for 300 min. The same GCaMP6 images scaled with a pseudocolour bar from one representative pollen grains are shown in Fig. 4a for wild-type and Fig. 4b for *osca2.1/2.2*.

Supplementary Video 3 The impaired Ca^2+^ oscillations prior to germination in *osca2.1/2.2* pollen grains are rescued under hypo-osmolarity media. Pollen grains from wild type and *osca2.1/2.2* plants expressing the Ca^2+^ indicator GCaMP6 were placed on the hypo-osmotic (350 mOsm) germination medium, and GFP fluorescence images were taken every 30 s for 300 min. Similar GCaMP6 images scaled with a pseudocolour bar from representative wild-type and *osca2.1/2.2* pollen grains were shown in Fig. 5a and Fig. 5e, respectively.

Supplementary Video 4 Ca^2+^ oscillations prior to germination are attenuated under hyper-osmolarity media in both wild-type and *osca2.1/2.2* pollen grains, but more in *osca2.1/2.2*. Pollen grains from wild type and *osca2.1/2.2* plants expressing the Ca^2+^ indicator GCaMP6 were placed on the hyper-osmotic (680 mOsm) germination medium, and GFP fluorescence images were taken every 30 s for 300 min. Similar GCaMP6 images scaled with a pseudocolour bar from representative wild-type and *osca2.1/2.2* pollen grains were shown in Fig. 5b and Fig. 5f, respectively.

### Source data

Source Data Fig. 1

Source Data Fig. 2

Source Data Fig. 3

Source Data Fig. 4

Source Data Fig. 5

Source Data Extended Data Fig. 2

Source Data Extended Data Fig. 3

Source Data Extended Data Fig. 4

Source Data Extended Data Fig. 5

Source Data Extended Data Fig. 6

Source Data Extended Data Fig. 7

Source Data Extended Data Fig. 8

Source Data Extended Data Fig. 9

Source Data Extended Data Fig. 10

## Data Availability

Information on OSCA T-DNA insertion lines and PCR primers and vectors are provided in Supplementary Tables 1 and 2. DNA and RNA gel images presented in Extended Data Fig. 3a,b are included in Supplementary Fig. 1. All other data and materials supporting the findings of this study are available in the main text or the supplementary materials. Source data are provided with this paper.
