# Supplementary material for: Osmosensor-mediated control of Ca2+ spiking in pollen germination
Source: Nature. 2024 May 22;629(8014):1118–25. doi: 10.1038/s41586-024-07445-6 (PMC11136663; doi:10.1038/s41586-024-07445-6)
Supplement: Supplementary file 1 — This file contains Supplementary Figure 1: Gel source data; Supplementary Table 1: OSCA T-DNA insertion lines and primers used for genotyping; and Supplementary Table 2: PCR primers and vectors. [file 41586_2024_7445_MOESM1_ESM.pdf]

nature portfolio

https://doi.org/10.1038/s41586-024-07445-6

Supplementary information

Osmosensor-mediated control of Ca^{2+} spiking in pollen germination

In the format provided by the authors and unedited

Supplementary Figure 1. Gel source data.

Boxes indicate cropping for representative images.
Note: Extended Data Fig. 3a and Extended Data Fig. 3b showed the whole gel scan.

Extended Data Fig. 3a

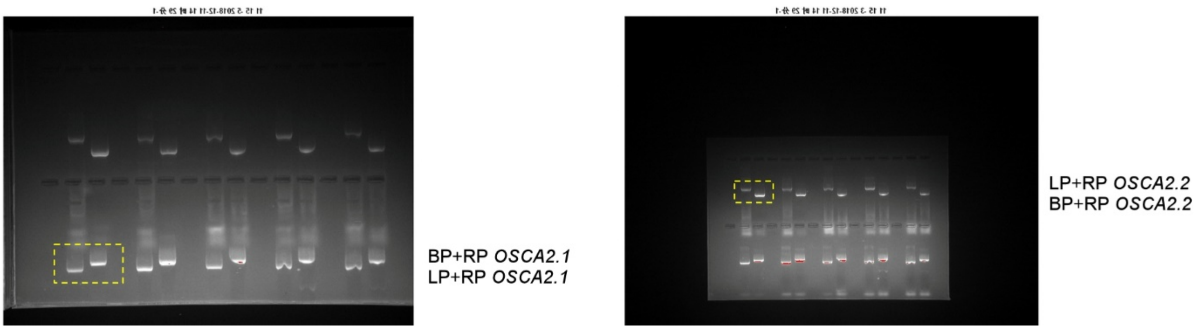

Extended Data Fig. 3b

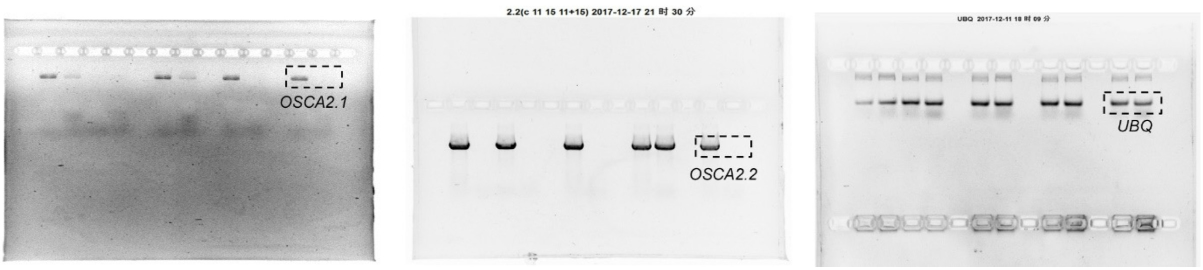

Supplementary Table 1. OSCA T-DNA insertion lines and primers used for genotyping.

AGI code	OSCA	Mutant ID	Amplified DNA size (bp)	Primer (5' to 3')
At4g04340	1.1-2	SAIL_607_F09	W: ~1107<br>T: 500-800	F: TAACCATTCAGTTGGGTTTCG<br>R: ATTGGACAAACAACGAGTTGG
At4g22120	1.2	GABI_743H03	W: ~1185<br>T: 595-895	F: GCCTTTAAGGTTCAGGTTTGG<br>R: TTGCTACCATTGAAGGGATTG
At1g11960	1.3	SALK_129246	W: ~1207<br>T: 576-876	F: GAATCAGTCTTTTTCCTCGGG<br>R: TTTTATCGGTATCGCTACCCC
At1g62320	1.4	SALK_135698C	W: ~1115<br>T: 543-843	F: AGGCGATGTGCATTACAAATC<br>R: AATGTCGAACGTGGATCAGAC
At3g21620	1.5	WISCDSLOXHS102_10D	W: ~1144<br>T: 589-889	F: AATGGATCCTGGAACCATAGG<br>R: ACTGCGAGGATCAATGTCAAC
At4g15430	1.6	SALK_058348	W: ~1132<br>T: 494-794	F: AAGATGAGGAGGAGGATCCAG<br>R: CTCACATTTTGCCAACTCCTC
At4g02900	1.7	SALK_050701	W: ~1133<br>T: 456-756	F: GCATTTGTTCAGTCTCTTGCC<br>R: AAATTTTGAGAAGGCCGATTC
At1g32090	1.8	SALK_044650C	W: ~1203<br>T: 514-814	F: GGTTTTTCCGTTAAGAAAGGTC<br>R: CCAGCTCTACTTCCTCTTGGG
At1g58520	2.1	SALK_049668C	W: ~807<br>T: 549-849	F: CACATCAATCAAGCACCG<br>R: GGCTCTTATCTGGAATCTGGTA
At1g10090	2.2	WISCDSLOX303H03	W: ~1431<br>T: 511-811	F: CTGCTTGGTTGACCTTAGCAG<br>R: AAGGAAATGCTGACCTGTTC
At3g01100	2.3	SALK_117250.55.50.x	W: ~1161<br>T: 607-907	F: ACTGTTCCCGGATACAAGAGG<br>R: TTACGTATGGCGAGAGAGCTC
At1g69450	2.4	SALK_058448	W: ~961<br>T: 446-746	F: CTTTCCTGCAACAGAGATTGC<br>R: ATTGCGTTGAAAAGGATTGAG
At3g54510	2.5	SALK_055548C	W: ~1084<br>T: 475-775	F: TTGAGTGCTGCTGTGTTTGAG<br>R: CTTGATGCTCTCGTCCTCATC
At1g30360	3.1	SALK_004685	W: ~1121<br>T: 512-812	F: TACGTGTGGTGAACAAGCAAG<br>R: TTCCAAGTGGTAATGTTTGGC
At4g35870	4.1	SAIL_670_B02	W: ~1008<br>T: 448-748	F: AAACAGCACGACATTGGAAAC<br>R: TTCAATCTCACCATCTTTGCC

Forward (F) and reverse (R) primers were designed in the up- and down-stream regions of T-DNA insertion. The primers for T-DNA borders are 5’- ATTTTGCCGATTTCGGAAC-3’ (LBb1.3) for SALK, 5’- TCTGAATTTCATAACCAATCTCG-3’ for SAIL, 5’-TAATAATGTGTGAGTAGTTCCCAGA-3’ for WiscDsLox, and 5’-ATATTGACCATCATACTCATTGC-3’ for GABI-Kat (GK).

Supplementary Table 2. PCR primers and vectors.

Purpose	Primer name	Sequence (5'-3')	Destination vector
E.coli assay	OSCA1.1-cDNA_Fw	CACCATGGCTTCAGTACAAGATATTG	pDEST14, pcDNA3.2
E.coli assay	OSCA1.1-cDNA_Rev	CTAACAACTGTATGAAACAACAAT	pDEST14, pcDNA3.2
E.coli assay	OSCA1.2-cDNA_Fw	CACCATGGCGACACTTCAGGATA	pDEST14
E.coli assay	OSCA1.2-cDNA_Rev	TTAGACTAGTTTACCACTAAAGGG	pDEST14
E.coli assay	OSCA1.3-cDNA_Fw	CACCATGGCAACACTAGGAGATATTGG	pDEST14
E.coli assay	OSCA1.3-cDNA_Rev	TTACTTTGAGGGTGGTGAACG	pDEST14
E.coli assay	OSCA1.4-cDNA_Fw	CACCATGGCGACATTAGCAGATA	pDEST14
E.coli assay	OSCA1.4-cDNA_Rev	TCAAGTCTCCGGTTTCC	pDEST14
E.coli assay	OSCA1.5-cDNA_Fw	CACCATGGCGACACTAACCGAT	pDEST14
E.coli assay	OSCA1.5-cDNA_Rev	CTAAGTAAAGGTTTCAGCAGAG	pDEST14
E.coli assay	OSCA1.6-cDNA_Fw	CACCATGGCTACAATAAACGATATTG	pDEST14
E.coli assay	OSCA1.6-cDNA_Rev	TTAGGGTATTTCACGGCAG	pDEST14
E.coli assay	OSCA1.7-cDNA_Fw	CACCATGGCTTCAGTACAAGATATTG	pDEST14
E.coli assay	OSCA1.7-cDNA_Rev	CTAACAACTGTATGAAACAACAAT	pDEST14
E.coli assay	OSCA1.8-cDNA_Fw	CACCATGGCGACTCTACAAGACATAG	pDEST14
E.coli assay	OSCA1.8-cDNA_Rev	TCAATTGTAACGGTACTCATGC	pDEST14
E.coli assay, HEK imaging	*OSCA2.1*-cDNA_Fw	CACCATGGAGATATCAGCTCTTCTTAC	pDEST14, pcDNA3.2
E.coli assay, HEK imaging	*OSCA2.1*-cDNA_Rev	TCAGGACTTAGTGTCATGTTCT	pDEST14, pcDNA3.2
E.coli assay, HEK imaging	*OSCA2.2*-cDNA_Fw	CACCATGGATGTCTCAGCACTTTTA	pDEST14, pcDNA3.2
E.coli assay, HEK imaging	*OSCA2.2*-cDNA_Rev	CTATGTTCGTTTGTAGATCTCG	pDEST14, pcDNA3.2
E.coli assay	OSCA2.3-cDNA_Fw	CACCATGGCACTTCTGTCAGCACTTTTA	pDEST14
E.coli assay	OSCA2.3-cDNA_Rev	TCAAGGTGAGATATCTCTCGAG	pDEST14
E.coli assay	OSCA2.4-cDNA_Fw	CACCATGGCACTTCTGTCAGCACTTTTA	pDEST14
E.coli assay	OSCA2.4-cDNA_Rev	TCAAGGTGAGATATCTCTCGAG	pDEST14
E.coli assay	OSCA2.5-cDNA_Fw	CACCATGCTTTTGTCTGCACTTCTT	pDEST14
E.coli assay	OSCA2.5-cDNA_Rev	TCAAACCTCACTAAAAGAGAGAA	pDEST14
E.coli assay	OSCA3.1-cDNA_Fw	CACCATGGAGTTTGGATCTTTTCTT	pDEST14
E.coli assay	OSCA3.1-cDNA_Rev	TTAAACGCCTGCTATTGC	pDEST14
E.coli assay	OSCA4.1-cDNA_Fw	CACCATGGCAAATCGCAATTTTTCACC	pDEST14
E.coli assay	OSCA4.1-cDNA_Rev	TCATCTGTTGTTGTAAGTGTCC	pDEST14
E.coli assay	genomic-DNA_Fw	CACCAAAGGAATTAAGGACCGGTA	pDEST14
E.coli assay	genomic-DNA_Rev	CTGGGTTTAGCCAGAGGA	pDEST14
OSCA-GFP HEK	*OSCA2.1*-GFP HEK_Fw	CCGCTCGAGATGGAGATATCAGCTCTTC	pEGFP-N1
OSCA-GFP HEK	*OSCA2.1*-GFP HEK_Rev	GGAATTCGGGACTTAGTGTCATGTTCTT	pEGFP-N1
OSCA-GFP HEK	*OSCA2.2*-GFP HEK_Fw	CCGCTCGAGATGGATGTCTCAGCACTTTT	pEGFP-N1
OSCA-GFP HEK	*OSCA2.2*-GFP HEK_Rev	GGAATTCGTGTTCGTTTGTAGATCTCGG	pEGFP-N1
OSCA-YFP Arabidopsis	p*OSCA2.1*_Fw	CGGGATCCTGTTGAGGAATCAAAGAAAATTGT	pGWB540
OSCA-YFP Arabidopsis	p*OSCA2.1*_Rev	ATAAGAATGCGGCCGCCTTAATCTTAGTTCAACTCCTTC	pGWB540
OSCA-YFP Arabidopsis	*OSCA2.1*-YFP AT_Fw	ATAAGAATGCGGCCGCATGGAGATATCAGCTCTTCTTAC	pGWB540
OSCA-YFP Arabidopsis	*OSCA2.1*-YFP AT_Rev	CCGCTCGAGTGGGACTTAGTGTCATGTTCTTGACCAG	pGWB540
OSCA-YFP Arabidopsis	p*OSCA2.2*_Fw	CGGGATCCTGAAGAAGTTGGCCGAGAA	pGWB540
OSCA-YFP Arabidopsis	p*OSCA2.2*_Rev	ATAAGAATGCGGCCGCCTCACTATCTCACTTTTAGTTC	pGWB540
OSCA-YFP Arabidopsis	*OSCA2.2*-YFP AT_Fw	ATAAGAATGCGGCCGCATGGATGTCTCAGCACTTTTA	pGWB540
OSCA-YFP Arabidopsis	*OSCA2.2*-YFP AT_Rev	GATATCTTGTTCGTTTGTAGATCTCGG	pGWB540
GUS analysis	p*OSCA2.1*-GUS_Fw	CGGGATCCTGTTGAGGAATCAAAGAAAATTGT	pGWB533
GUS analysis	p*OSCA2.1*-GUS_Rev	CCGCTCGAGTGGGACTTAGTGTCATGTTCTTGACCAG	pGWB533
GUS analysis	p*OSCA2.2*-GUS_Fw	CGGGATCCTGAAGAAGTTGGCCGAGAA	pGWB533
GUS analysis	p*OSCA2.2*-GUS_Rev	GATATCTTGTTCGTTTGTAGATCTCGG	pGWB533
RT-PCR analysis	*OSCA2.1*-RT_Fw	TTTTCCTTGCATATCTCA	
RT-PCR analysis	*OSCA2.1*-RT_Rev	TGTAACTCTTCCATCTTTC	
RT-PCR analysis	*OSCA2.2*-RT_Fw	CAAGTCTGCCACCCATAA	
RT-PCR analysis	*OSCA2.2*-RT_Rev	CCTAATGGTCTACCGTGAT	
RT-PCR analysis	UBQ_Fw	TAAAAACTTTCTCTCAATTCTCTCT	
RT-PCR analysis	UBQ_Rev	TTGTCGATGGTGTCGGAGCTT	
