# Supplementary material for: Osmosensor-mediated control of Ca2+ spiking in pollen germination
Source: Nature. 2024 May 22;629(8014):1118–25. doi: 10.1038/s41586-024-07445-6 (PMC11136663; doi:10.1038/s41586-024-07445-6)
Supplement: Supplementary file 2 — Reporting Summary [file 41586_2024_7445_MOESM2_ESM.pdf]

nature portfolio

Corresponding author(s): Zhen-Ming Pei

Last updated by author(s): Mar 5, 2024

Reporting Summary

Nature Portfolio wishes to improve the reproducibility of the work that we publish. This form provides structure for consistency and transparency in reporting. For further information on Nature Portfolio policies, see our Editorial Policies and the Editorial Policy Checklist.

Statistics

For all statistical analyses, confirm that the following items are present in the figure legend, table legend, main text, or Methods section.

n/a	Confirmed	
☐	☒	The exact sample size (*n*) for each experimental group/condition, given as a discrete number and unit of measurement
☐	☒	A statement on whether measurements were taken from distinct samples or whether the same sample was measured repeatedly
☐	☒	The statistical test(s) used AND whether they are one- or two-sided<br>*Only common tests should be described solely by name; describe more complex techniques in the Methods section.*
☐	☒	A description of all covariates tested
☐	☒	A description of any assumptions or corrections, such as tests of normality and adjustment for multiple comparisons
☐	☒	A full description of the statistical parameters including central tendency (e.g. means) or other basic estimates (e.g. regression coefficient) AND variation (e.g. standard deviation) or associated estimates of uncertainty (e.g. confidence intervals)
☐	☒	For null hypothesis testing, the test statistic (e.g. *F*, *t*, *r*) with confidence intervals, effect sizes, degrees of freedom and *P* value noted<br>*Give P values as exact values whenever suitable.*
☒	☐	For Bayesian analysis, information on the choice of priors and Markov chain Monte Carlo settings
☒	☐	For hierarchical and complex designs, identification of the appropriate level for tests and full reporting of outcomes
☒	☐	Estimates of effect sizes (e.g. Cohen's *d*, Pearson's *r*), indicating how they were calculated

Our web collection on statistics for biologists contains articles on many of the points above.

Software and code

Policy information about availability of computer code

Data collection	MetaMorph 7.7 and MetaFluor 7.7 (Molecular Devices) ; Micro-Manger software; Zen 2012.
Data analysis	EXCEL16 software (Microsoft); SAS software (SAS Institute); MetaMorph 7.7 and MetaFluor 7.7; ImageJ (1.52g).

For manuscripts utilizing custom algorithms or software that are central to the research but not yet described in published literature, software must be made available to editors and reviewers. We strongly encourage code deposition in a community repository (e.g. GitHub). See the Nature Portfolio guidelines for submitting code & software for further information.

Data

Policy information about availability of data

All manuscripts must include a data availability statement. This statement should provide the following information, where applicable:

- Accession codes, unique identifiers, or web links for publicly available datasets
- A description of any restrictions on data availability
- For clinical datasets or third party data, please ensure that the statement adheres to our policy

□ Numerical source data giving rise to graphical representation for each data point for main and extended data figures are provided as source data. The information of OSCA T-DNA insertion lines and PCR primers and vectors are provided in Supplementary Tables 1 and 2. DNA and RNA gel images presented in Extended Data Fig. 3a,b are included in Supplementary Fig. 1. All other data and materials supporting the findings of this study are available in the main text or the supplementary materials. Source data are provided with this paper.

Human research participants

Policy information about studies involving human research participants and Sex and Gender in Research.

Reporting on sex and gender	n/a
Population characteristics	n/a
Recruitment	n/a
Ethics oversight	n/a

Note that full information on the approval of the study protocol must also be provided in the manuscript.

Field-specific reporting

Please select the one below that is the best fit for your research. If you are not sure, read the appropriate sections before making your selection.

☒ Life sciences ☐ Behavioural & social sciences ☐ Ecological, evolutionary & environmental sciences

For a reference copy of the document with all sections, see nature.com/documents/nr-reporting-summary-flat.pdf

Life sciences study design

All studies must disclose on these points even when the disclosure is negative.

Sample size	No statistical methods were used to predetermine sample size. Sample size was chosen as large as possible and in accordance with previous established protocols in the field and references cited in the Methods section.
Data exclusions	No data were excluded from the analyses.
Replication	The recordings and parameters were reproducible across multiple days and batches, which was dependent on the experiments. For instance, in vitro pollen germination experiments were repeated from several months to several years. Ca2+ imaging experiments were repeated from several weeks to several months. In vivo pollen germination was repeated several days to several weeks.
Randomization	All samples were allocated randomly into experimental groups.
Blinding	Blinding was not relevant to this study; Key experiments, such as E coli experssion, pollen germination, Ca2+ imaging, were repeated by several researchers throughout several years.

Reporting for specific materials, systems and methods

We require information from authors about some types of materials, experimental systems and methods used in many studies. Here, indicate whether each material, system or method listed is relevant to your study. If you are not sure if a list item applies to your research, read the appropriate section before selecting a response.

Materials & experimental systems

n/a	Involved in the study	
☒	☐	Antibodies
☐	☒	Eukaryotic cell lines
☒	☐	Palaeontology and archaeology
☒	☐	Animals and other organisms
☒	☐	Clinical data
☒	☐	Dual use research of concern

Methods

n/a	Involved in the study	
☒	☐	ChIP-seq
☒	☐	Flow cytometry
☒	☐	MRI-based neuroimaging

Eukaryotic cell lines

Policy information about cell lines and Sex and Gender in Research

Cell line source(s)	HEK293 cells were purchased from vendors.

Authentication	No further authentication of the cell line was done after purchasing.
Mycoplasma contamination	No further test for mycoplasma contamination of the cell line was done after purchasing.
Commonly misidentified lines (See ICLAC register)	The HEK293 cell line used in this study is not amongst the misidentified cell lines.
